# Development and mechanisms of photo-induced molecule junction device

**DOI:** 10.1515/nanoph-2023-0921

**Published:** 2024-03-06

**Authors:** Xin Sun, Ran Liu, Sneha Kandapal, Bingqian Xu

**Affiliations:** Single Molecule Study Laboratory, College of Engineering and Nanoscale Science and Engineering Center, University of Georgia, Athens, GA 30602, USA

**Keywords:** optoelectronic, single molecule junctions, photo-induced switch, charge transport, molecular electronics, photoemission

## Abstract

The utilization of single molecule electronic devices represents a significant avenue toward advancing next-generation circuits. Recent investigations have notably augmented our understanding of the optoelectronic characteristics exhibited by diverse single molecule materials. This comprehensive review underscores the latest progressions in probing photo-induced electron transport behaviors within molecular junctions. Encompassing both single molecule and self-assembled monolayer configurations, this review primarily concentrates on unraveling the fundamental mechanisms and guiding principles underlying photo-switchable devices within single molecule junctions. Furthermore, it presents an outlook on the obstacles faced and future prospects within this dynamically evolving domain.

## 1 Introduction

In the last two decades, molecular electronics (ME) has witnessed remarkable growth, largely attributed to Aviram and Ratner’s proposition of employing single molecules within electronic circuits [1]. ME, an interdisciplinary domain encompassing physics, chemistry, biology, and nanoengineering, has emerged as a pivotal arena investigating charge dynamics and energy transformations at the molecular scale. Molecular junctions (MJs), minute structures linking molecules between two conductive electrodes, have garnered considerable attention as researchers aim to decipher and efficiently control electron transport through them [2], [3], [4], [5], [6], [7], [8].

Recent advancements in experimental methodologies for fabricating and assessing molecular junctions have facilitated exploration beyond conventional charge transport. Intriguing transport phenomena within MJs have been observed, including the transistor effect [9], [10], [11], phenomena tied to thermal aspects [12], [13], [14], conductance modulation [15], [16], [17], photoswitching [18], [19], [20], [21], and spintronics [22], [23], [24].

This review focuses on optoelectronic phenomena within MJs, a relatively less explored but crucial facet driving the field forward. The interaction between light and MJs, particularly under UV and visible illumination, has gained increasing attention for its potential in diverse applications such as photovoltaics [25], [26], [27], light-emitting diodes [21], [28], chemical and bio-detection [29], [30], [31], and plasmonic [32], [33], [34], [35], [36]. Light serves as a beneficial external stimulus for MJs, providing advantages like remote control, swift response, a wide spectrum of wavelengths, and non-invasive influence. Illuminated MJs can induce novel transport effects stemming from various interactions – between light and molecules, light and metal electrodes, and light-triggered alterations in the local environment. These alterations encompass photoisomerization, heating, surface plasmon generation, and intense electric fields [37], [38], [39], [40], resulting in augmented molecular conductance, generation of high-energy hot-carriers, photoemission, and structural changes [41], [42], [43].

Self-assembled monolayers (SAMs) have emerged as a versatile and widely explored component in various scientific domains. SAMs, composed of spontaneously organized molecules forming a single layer, exhibit unique properties that have found applications in diverse fields such as surface modification [44], [45], molecular electronics and sensing [46], [47]. Notably, SAMs have been extensively utilized in the realm of solar cells, demonstrating their potential to enhance device performance through controlled interfaces and improved charge transport properties [48], [49], [50]. While acknowledging the significance of SAMs in solar cell applications, it is imperative to clarify that this review paper maintains a specific focus on the intriguing and nuanced domain of single-molecule photovoltaics. Our exploration delves into the distinctive aspects of individual molecules in generating and manipulating photovoltaic responses. This deliberate emphasis allows us to contribute to the evolving understanding of the fundamental principles governing single-molecule behavior in photovoltaic applications.

**Figure 1:**
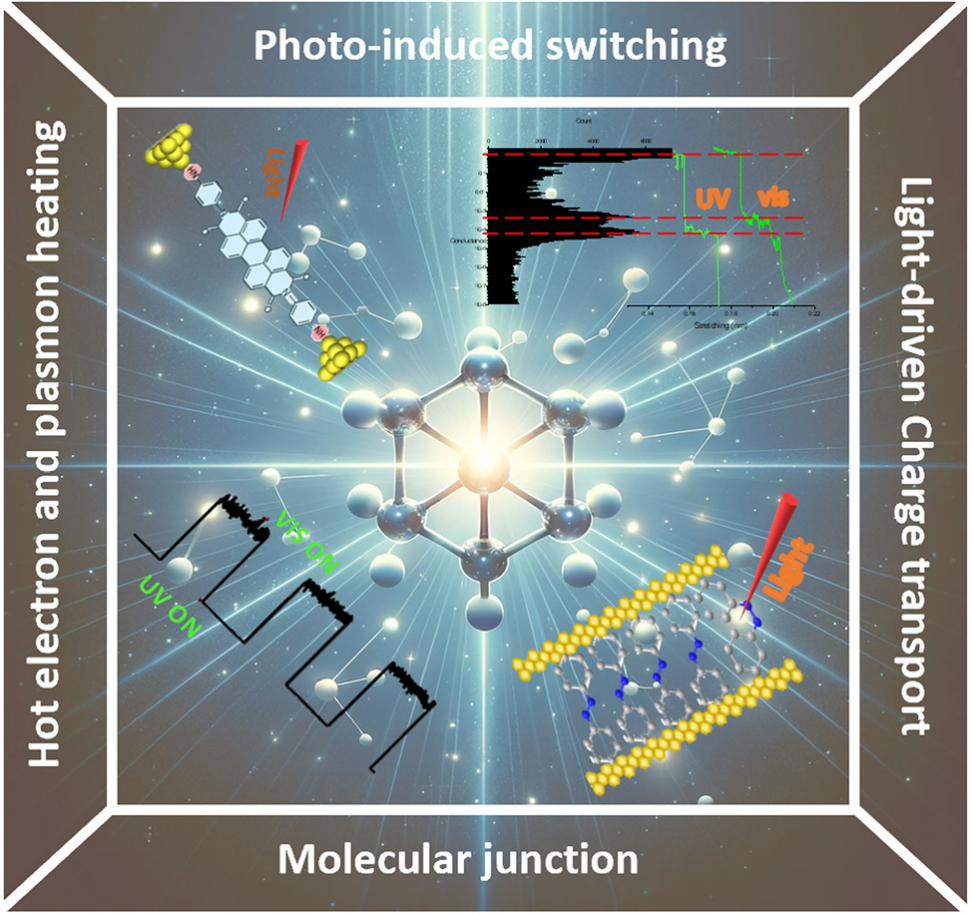
Schematic overview of the subjects addressed in this overview.

As shown in Figure 1, this review aims to offer a comprehensive overview of recent advancements in optoelectronic phenomena within illuminated molecular junctions, emphasizing light-driven charge transport and optical sensing within these structures. Encompassing both single molecular junctions and SAM junctions, this article’s structure is organized as follows: initial sections introduce various techniques for constructing and analyzing single molecular devices, followed by discussions on recent findings regarding conductance alterations in single molecules. Subsequently, significant progress in studying photochromic molecules and SAM junctions is highlighted. Finally, the review explores existing challenges and emerging opportunities in this evolving field.

## 2 Techniques to study the optoelectronic properties of single molecular junctions

Molecular electronics has two primary objectives: understanding the charge transport properties within molecule-based devices and exploring the intrinsic properties of molecules. An essential step in accomplishing these aims involves establishing electrical connections between molecules and external electrodes. Electronic junctions, classified by the number of connected molecules, fall into two categories: single-molecule junctions and ensemble molecular junctions [38], [51], [52]. Single-molecule junctions entail positioning a few or even just one molecule between two electrodes, while ensemble molecular junctions typically comprise a molecular monolayer housing numerous molecules.

Considerable research endeavors have concentrated on establishing dependable molecular junctions. Techniques such as mechanically controllable break junctions (MCBJ) and scanning tunneling microscopy break junctions (STM-BJ) [8], [53], [54], [55] have provided insights into the intricate charge transport mechanisms at the molecular level, which are indispensable for advancing the field of molecular electronics. To further the practical applications of molecule-based devices, considerable attention has been devoted to methods enhancing the device yield of ensemble molecular junctions. Among these approaches, conductive atomic force microscopy (C-AFM) technology has emerged as a noteworthy method [56], [57]. This focus on ensemble molecular junctions is key to transitioning molecular electronics from theoretical research to practical, real-world applications. This emphasis on ensemble molecular junctions holds the key to transitioning molecular electronics from a theoretical realm to practical, real-world applications.

### 2.1 Scanning tunneling microscopy break junctions (STM-BJ)

Scanning probe microscopies, particularly scanning tunneling microscopy (STM) and conductive atomic force microscopy (C-AFM), have been pivotal in transforming single-molecule investigations since the 1980s. STM excels in combining spatially resolved tunneling spectroscopy with high-resolution imaging [58].

**Figure 2:**
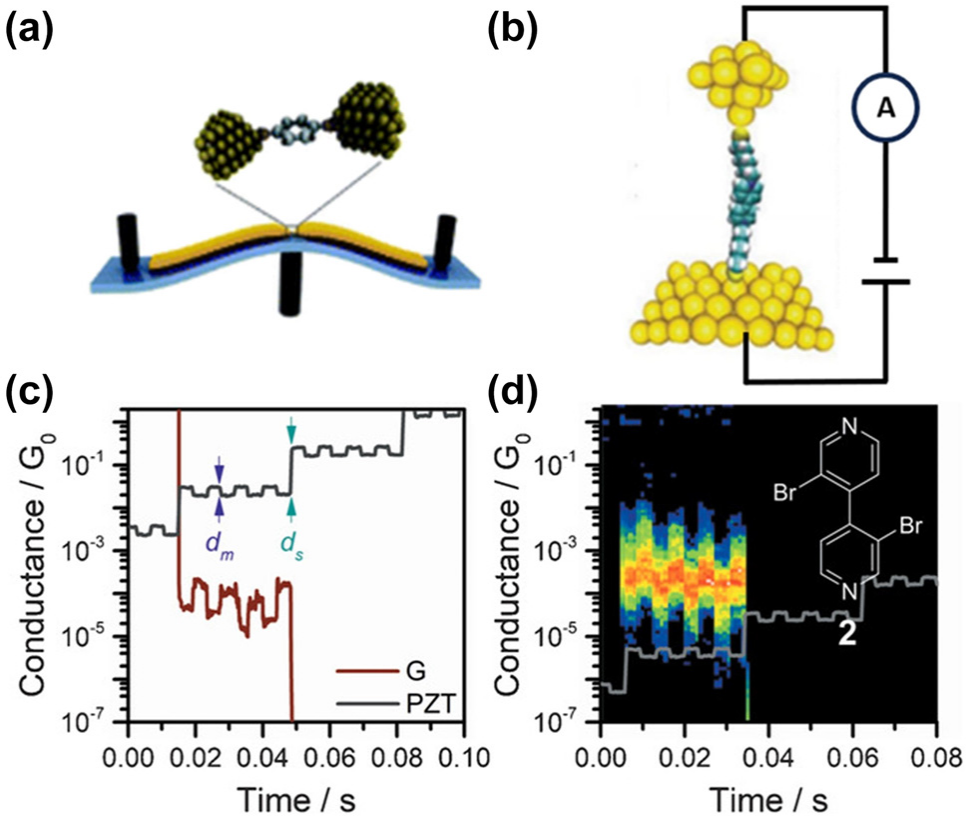
Schematic and modulation measurement of MCBJ and STMBJ technology. (a) Schematic of MCBJ. (b) Schematic of STM-BJ. (c) Conductance versus time trace and modulation 2d density maps (d) under piezo modulation for dipyridyls compound 2. Inset shows the structure of the compound. Figure (a, c, d) reprinted with permission from Refs. [59], [60].

The STM-BJ method enables swift and repetitive creation of metal–molecule–metal junctions by adjusting the gap between an STM probe tip and a metal substrate adorned with adsorbed molecules (Figure 2b) [7], [8]. Precise control over the STM tip’s movement is achieved using a piezoelectric transducer. In typical experiments, the molecules under study possess two end groups attaching to both the substrate and tip electrodes. As the tip approaches the substrate, molecules can bridge the gap between the STM tip and substrate electrodes. Upon the subsequent tip retraction from the substrate, the number of bridged molecules diminishes until a single-molecule junction is established. However, this method faces limitations in determining molecule concentrations beyond the millimolar range where single-molecule saturation occurs. Additionally, electrode bridging might occur in varying configurations, posing challenges in identifying the junction configuration. To address this, continuous-stretching and stretch-holding modulation techniques have been employed to gain deeper insights into molecular configurations [61], [62], [63], [64].

Researchers, such as Ismael et al., have investigated the junction switching of functionalized dipyridyl compounds by employing rectangular mechanical manipulation of STM piezoelectric transducer movement [59]. Their findings revealed an approximate one order of magnitude change in conductance for these compounds in response to piezo modulation (Figure 2c and d). This alteration in conductance was linked to the varying geometries observed at the Au–N interface.

### 2.2 Mechanically controllable break junction (MCBJ)

A fundamental technique in molecular electronics, the mechanically controllable break junction (MCBJ) method, initially introduced by Moreland et al. and further refined by Reed et al., stands as a pivotal approach for creating nanogap electrodes within the nanometer scale, significantly impacting the field [65], [66]. The typical setup of an MCBJ, as depicted in Figure 2a, involves a notched metallic wire mounted on a flexible substrate known as a bending beam [60]. This beam, anchored at both ends, fractures upon bending by applying force at its center with a pushing rod, resulting in the formation of two opposing nanoelectrodes. To ensure electrode cleanliness, this fracture process typically occurs under conditions of high vacuum and low temperature.

The MCBJ technique offers precise control over the gap size between two tip-shaped nanoelectrodes, facilitated by vertical movement of a push rod via a piezoelectric motor or actuator. Reversing the bending of the substrate allows for re-approaching the electrode surfaces. This technology holds significant advantages for molecular electronics owing to its unique benefits. Integration with complementary systems, such as a Raman spectrometer, enables the collection of molecular fingerprint data. Notably, the break junction’s drift rate can be minimized to 0.2 pm h−1, and its suitability for single molecule measurements is notable, given the scalability of the electrodes to molecular dimensions [67].

**Figure 3:**
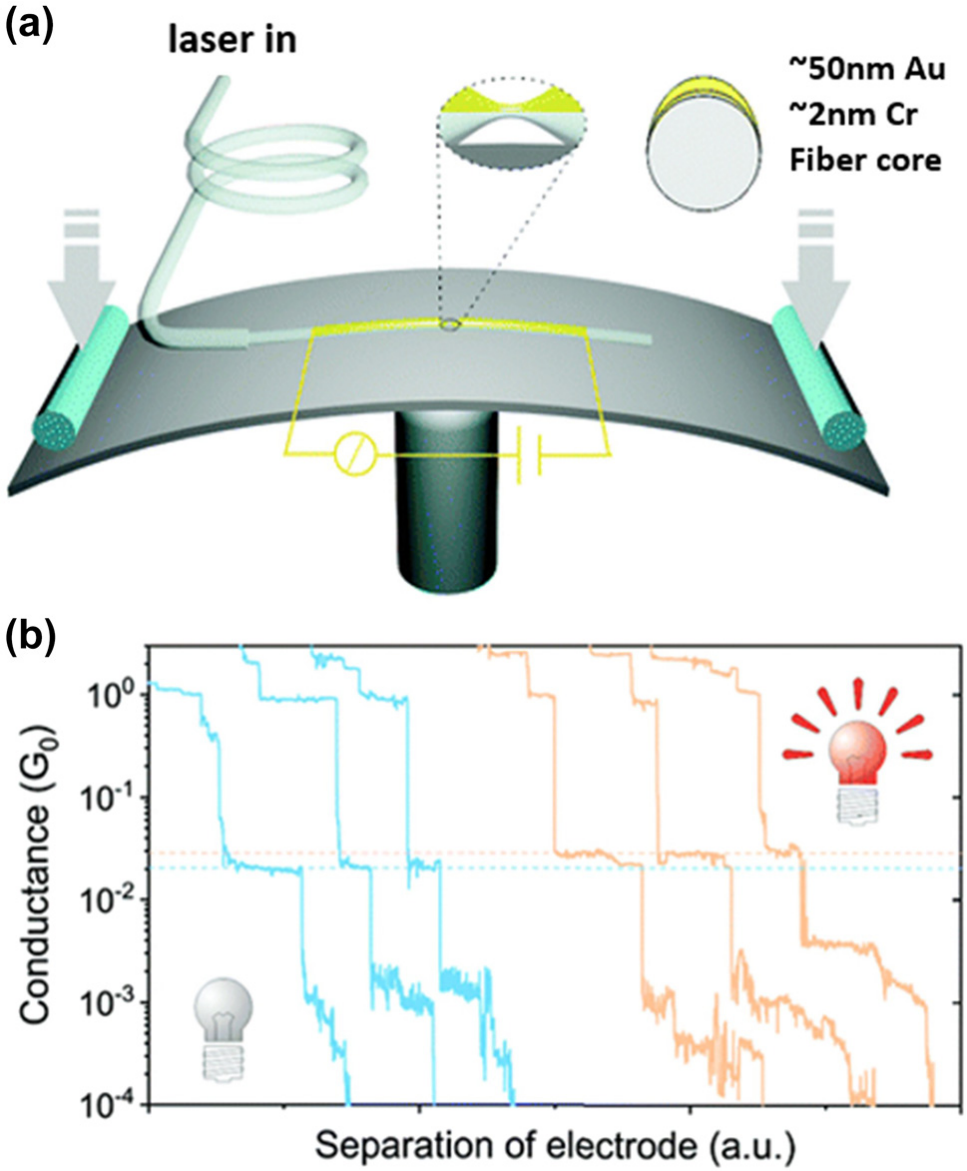
Illustraion of F-BJ experiment. (a) Schematic of F-BJ setup. The inset shows the suspended fiber/Cr/Au bridge. (b) Conductance traces of single-imidazole junction measured under the absence (sky blue) and presence (sand) of light illumination. Figure (a, b) reprinted with permission from Ref. [68].

Zhao et al. developed an optical fiber-based break junction (F-BJ) technique based on traditional MCBJ method [68]. This work provides a robust tool for tuning the optoelectronic performance of single-molecule devices *in situ*, and the observation of the interaction between single molecules and fiber transmitted light. In the modified platform, a metal-coated tapered optical fiber is fixed on a flexible substrate, and this fiber serves as both the optical waveguide and metal electrodes after it breaks (Figure 3a). Under the light illumination, the conductance of single-imidazole junction increased from 2.1 × 10^−2^ G_0_ to 2.6 × 10^−2^ G_0_ (Figure 3b), proving the stability and reliability of the system.

### 2.3 Conductive atomic force microscopy technology (C-AFM)

Atomic force microscopy (AFM), although akin to STM, operates on a different principle. Unlike STM’s reliance on current to control tip positioning, AFM utilizes force for this purpose [57], [69]. The conductance properties of molecules often lack precise definition, leading to uncertainty in the STM probe’s position concerning the molecules, occasionally causing penetration into self-assembled monolayers (SAMs). In contrast, AFM allows precise control in both noncontact and contact modes through independent feedback signals.

**Figure 4:**
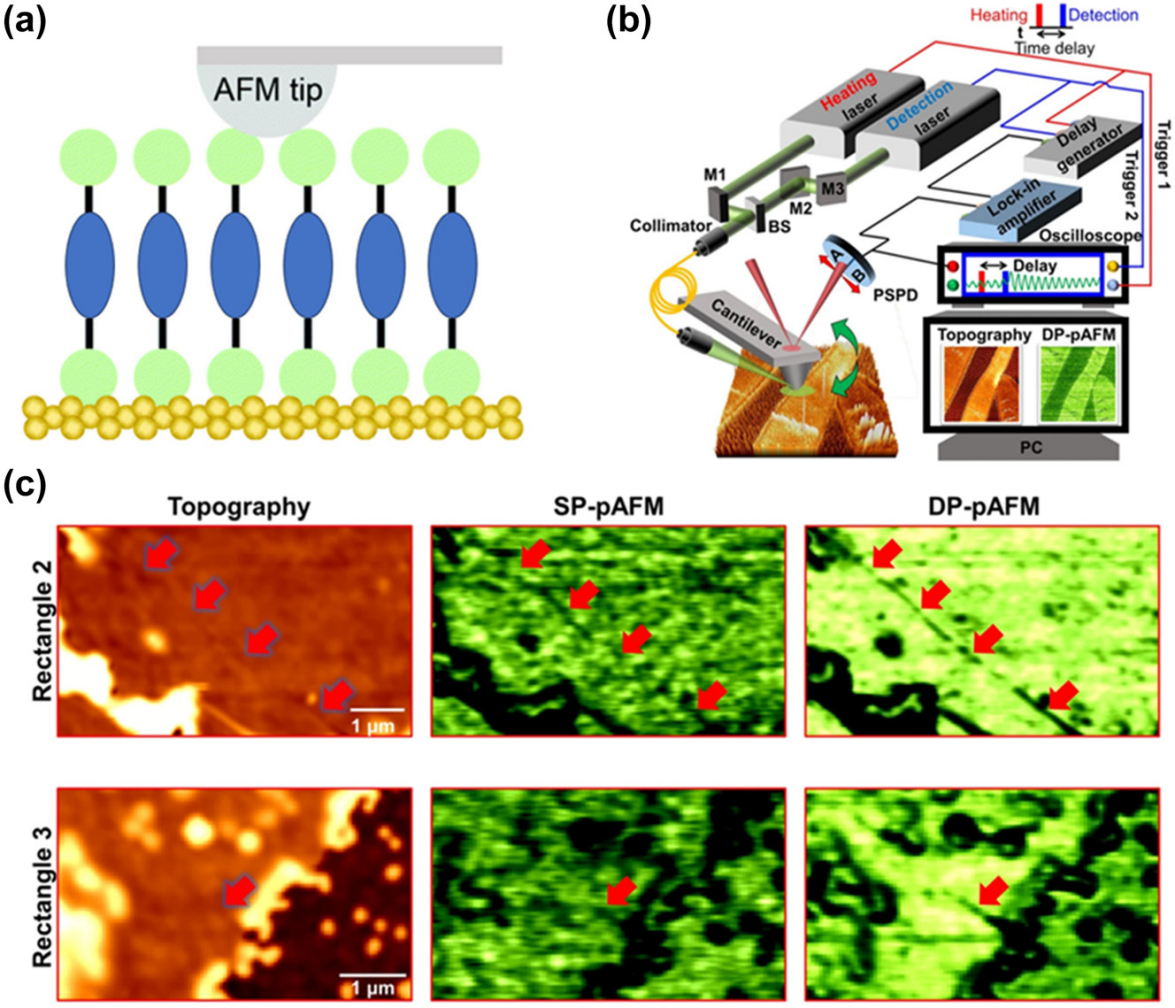
Schematic and real-time images of AFM and DP-pAFM. (a) Schematic of C-AFM. (b) Schematic diagram of DP-pAFM. (c) Zoomed-in images of nanosized cracks in small-molecule organic semiconductor film. Red arrows indicate a very fine crack. Figure (b, c) reprinted with permission from Ref. [70].

An inherent limitation of AFM is its inability to directly assess the electrical properties of molecules. To address this, the AFM probe tip is coated with a metallic layer, enabling the construction of a metal-self-assembled monolayers-metal junction when the C-AFM tip contacts the molecular layer. This depicted approach in Figure 4a facilitates simultaneous measurement of both electrical and mechanical properties of molecules [71], [72]. While C-AFM offers lower spatial resolution compared to STM due to a larger probe tip, it remains a valuable tool for constructing ensemble molecular junctions and investigating the interplay between charge transport and molecular conformations [73], [74]. Several modified AFM techniques, such as photo-induced force microscopy (PiFM), photothermal atomic force microscopy (PT-AFM), and photoactivated atomic force microscopy (pAFM), have been designed to enhance resolution and contrast [75], [76], [77].

Innovative methods continue to emerge within AFM. Park et al. introduced the dual-pulse photoactivated AFM (DP-pAFM) for high-resolution morphological and optical analysis of materials (Figure 4b) [70]. This technique enhances image contrast and sensitivity while minimizing power usage to reduce potential sample or cantilever tip damage. The method involves two synchronized lasers producing separate beams, merged and channeled onto the sample through a single-mode fiber. Sequential heating beneath the cantilever tip generates distinct oscillations, enabling precise mapping of optical structures in small-molecule semiconductor films. This approach successfully revealed nanoscale cracks in the small-molecule organic semiconductor ink, FlexOS film, which were challenging to discern in AFM topographic and pAFM images (Figure 4c).

## 3 Mechanisms for switching conductance in single molecule junctions

Over the last decade, the strategic manipulation of charge transport through molecules via optical methods has become integral to advancing molecular electronics. Recent explorations have centered on molecular junctions stimulated by external light, employing various experimental setups and theoretical frameworks. The primary aim of these investigations is to gain fundamental insights into the charge transport phenomena correlated with photon irradiation. This section focuses on scrutinizing several pivotal transport behaviors observed in molecular junctions under illumination.

### 3.1 Changes in molecular configuration triggered by light irradiation

The exposure of photochromic molecules like dihydroazulene and triphenylmethane derivatives to illumination induces structural transformations in these compounds [78], [79], [80], [81]. Recent investigations have unveiled that the conductance within molecular junctions hosting these photochromic molecules alters upon exposure to light. Photochromic systems fall into two categories: P-type and T-type. P-type systems, typified by diarylethenes, undergo a transition from one state to another upon light exposure, persisting in the altered state even in darkness or when heated. Conversely, T-type systems, including azobenzenes and dimethyldihydropyrenes (DHP), have the ability to thermally revert to their original photoisomeric forms upon heating.

**Figure 5:**
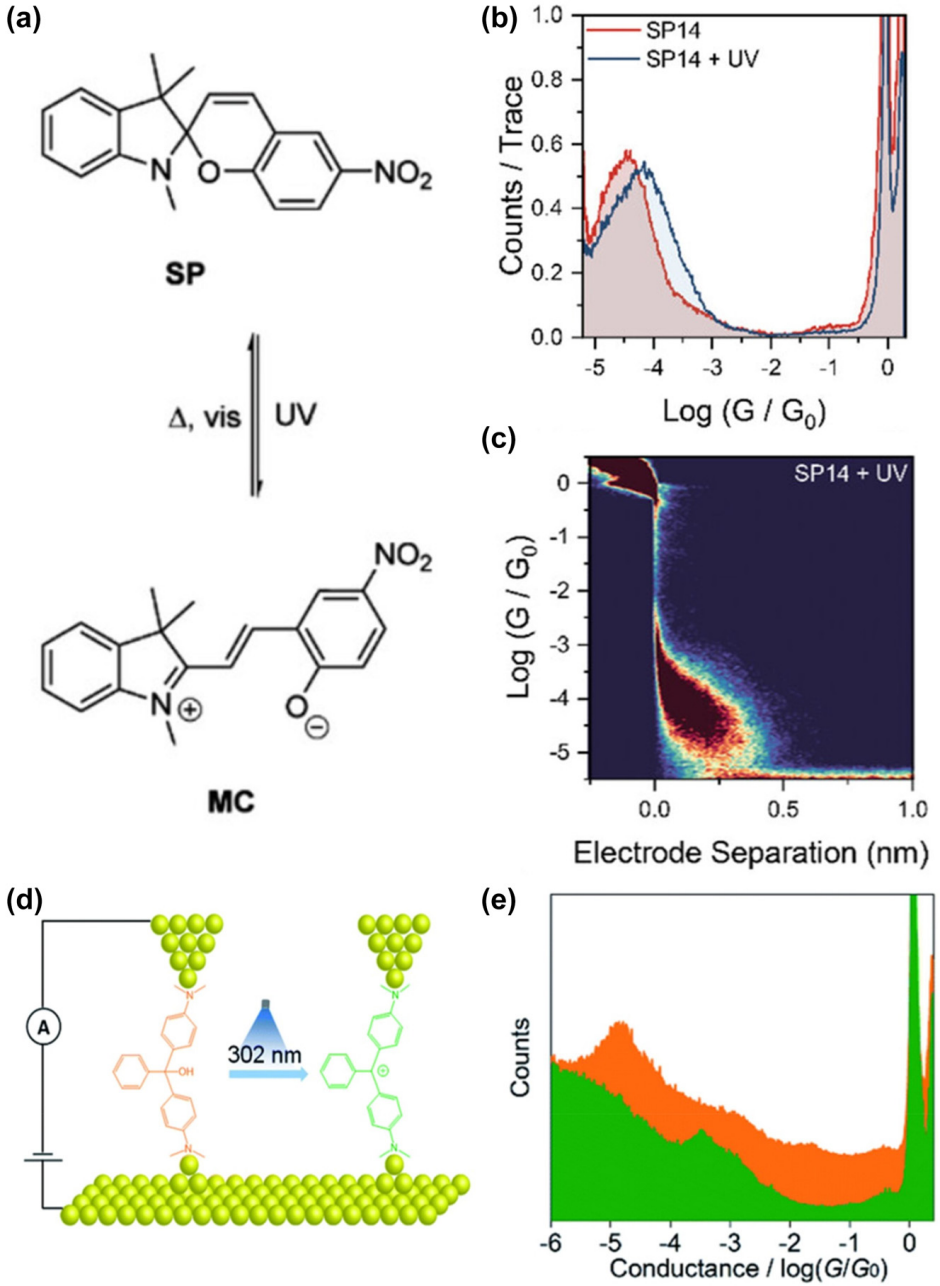
Stucture of SP/MC system and conductance switching phenomenon. (a) Scheme of the photoisomerization of the spiropyran to merocyanine. (b) Comparison of conductance histograms of SP14 before and after *in situ* irradiation with UV light. (c) 2D density map for SP14 after irradiation. (d) Schematic of the STM-BJ setup of MGOH molecule and photo-induced carbocation. (e) 1D conductance–displacement histogram results constructed from thousands of individual traces for MGOH (yellow) and carbocations (green). Figure (a–e) reprinted with permission from Refs. [82], [83].

In a study conducted by Jago et al., the STM-BJ method was combined with UV irradiation (385 nm) to explore photo reactions within a Spiropyran (SP)/Merocyanine (MC) system (Figure 5a) [83]. Their experiments under UV irradiation unveiled a transition from a less-conductive spiropyran form to a more efficient-conducting merocyanine structure (Figure 5b and c). Theoretical analyses suggested that the observed increase in conductance stems from torsional adjustments within the terphenyl-like backbone. These adjustments enhance orbital overlap between the anchor group and the central functional core within the junction.

In a separate investigation, Bei et al. employed the STM-BJ technique to observe photo-induced carbocation-enhanced charge transport in malachite green leuco hydroxide (MGOH) junctions [82]. Under 302 nm UV light (Figure 5d), MGOH transforms into malachite green carbocations, inducing a minor structural alteration in the central carbon atom from sp3 to sp2 hybridization. This transformation significantly increased the single-molecule conductance by a factor of 34 (Figure 5e). The reduction of the HOMO-LUMO gap and enhanced transmission near the Fermi levels were identified as pivotal factors contributing to the observed photo-induced carbocation-enhanced charge transport in MGOH upon carbocation formation.

### 3.2 Photon-assisted electron transport and hot electron

Illuminated molecular junctions exhibit enhanced conductance, attributed to a plasmon-induced electric field in the nanogap, following the Tien–Gordon model of photon-assisted tunneling or through hot electron generation [84]. Surface plasmons concentrate light within metallic nanogaps, intensifying the electromagnetic field and influencing conductance in single molecular junctions (MJs). The generation of surface plasmons induces a rectified dc current in MJs, underscoring their role in conductance enhancement [85], [86].

**Figure 6:**
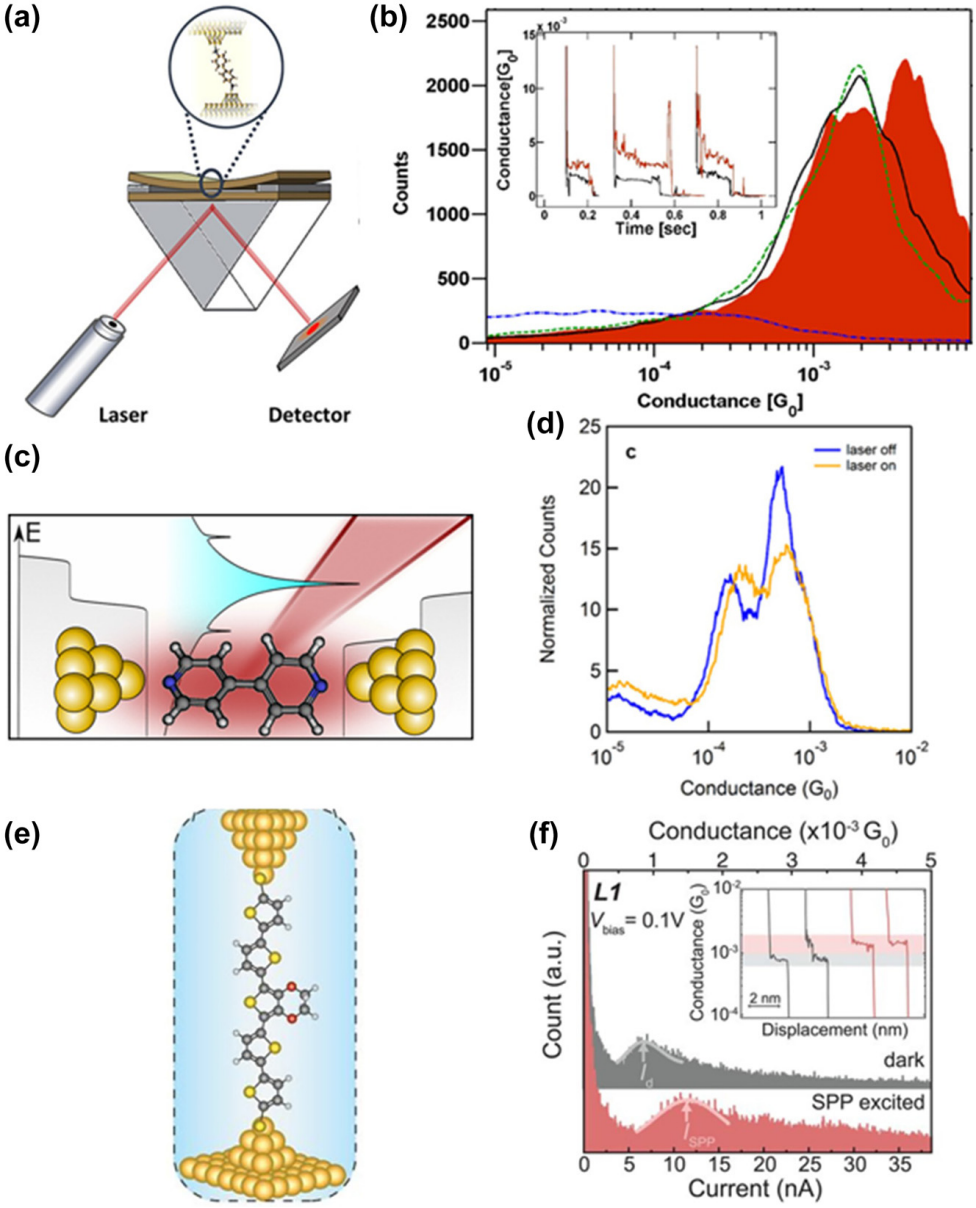
Hot-electron transport through DAF molecular junction. (a) SBJ setup for single-molecule conductance measurements. (b) 1D histogram of DAF at 781 nm without illumination (black solid line), with p-polarized light for which plasmons are created (red colored area), and with s-polarized light for which no plasmons should be created (green dashed line). Inset: representative conductance traces without (black) and with (red) laser illumination. (c) Schematic of illuminated 4,4′-bipyridine (BP) junction in the low-conducting geometry. (d) 1D conductance histograms of BP junctions for dark (laser off) and illuminated (laser on) environment. (e) Structure of the hot-carrier energy distribution experiment. (f) Current and conductance histograms of complex of quaterthiophene (T4) and tetracyanoethylene (TCNE) SMJs from more than 2000 traces of dark (gray) and SPP-excited (red) measurements at a *V*_bias_ of 0.1 V. Inset shows representative conductance traces. Figure (a–e) reprinted with permission from Refs. [43], [84], [87].

Vadai et al. conducted experiments on a single MJ made of 2,7-diaminofluorene (DAF) using a squeezable break junction (SBJ) technique under laser irradiation [87]. The SBJ setup, comprising two gold-coated glass slides with a controllable gap (Figure 6a), revealed that only p-polarized light elicited surface plasmons in the gap, thereby increasing conductance, unlike s-polarized light (Figure 6b). This rise in conductance primarily stemmed from the plasmon-induced oscillating electric field, as the plasmon energy was lower than the DAF’s HOMO-LUMO gap. The Tien–Gordon model, considering electron and electromagnetic field interactions within the molecular bridge, proposes enhanced electron transport via photon absorption.

Another study centered on 4,4′-bipyridine molecular junctions suggested that hot electron transport, where electrons absorb photons and experience prolonged relaxation time, significantly contributes to light-induced conductance enhancement (Figure 6c and d) [84]. However, the Tien–Gordon model holds relevance only if hot electrons exhibit a shorter lifetime than charge transfer time or if no light absorption occurs on the electrodes. Reddy et al. recently integrated a gold thin film plasmonic nanosurface into a molecular junction setup [43]. This experiment trapped single molecules between a gold film g supporting surface plasmon polaritons (SPPs) and a gold STM tip (Figure 6e). Laser illumination triggered surface plasmons, substantially augmenting the molecular junction current (Figure 6f). This observation, demonstrating polarization-dependent excitation, suggests Landau damping as a key mechanism for generating hot carriers in the junction. This approach opens novel avenues for exploring nanophotonic and plasmonic devices.

### 3.3 Plasmon-induced reaction

Hot electron transport emerges as a primary mechanism for optically induced conductance enhancement in single molecule junctions (SMJs). This process involves hot electrons transferring to molecule orbitals (MOs) through inelastic tunneling, generating transient negative ion states and triggering plasmon-induced reactions [88], [89], [90], [91].

**Figure 7:**
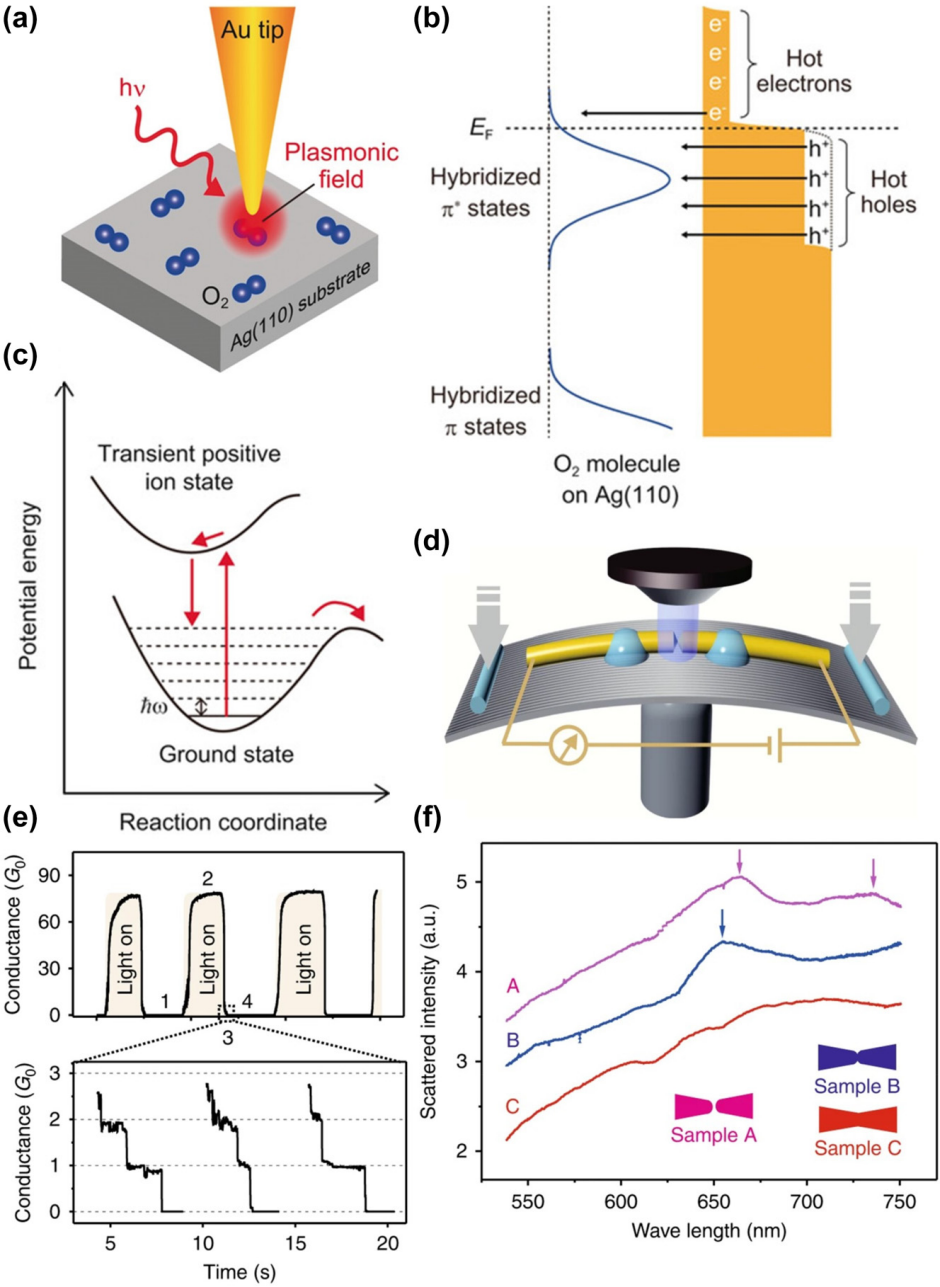
Plasmon-induced reaction and energy model of O2 molecule. (a) Schematic setup of plasmon-induced dissociation of a single O2 molecule in the nanogap between the Au tip and the Ag(110) substrate. (b) Schematic of the plasmon-induced excitation process O_2_ molecules. Hot carriers (holes and electrons) are transferred to the strongly hybridized π* states of the adsorbed O2 molecules. (c) Illustrations of the potential-energy surface for the plasmon-induced dissociation of the O2 molecule induced by the hot-hole transfer to the occupied π* states. Figure (a–f) reprinted with permission from Refs. [92], [93].

Kazuma et al. investigated the plasmon-induced dissociation of O_2_ molecules chemisorbed on Ag(110) (Figure 7a) [92]. Real-time information from STM confirmed that dissociation resulted from localized surface plasmons (LSP) rather than photon or thermal processes. Theoretical studies indicated that when a hot-hole transfers to the occupied π* states of the O2 molecule, it acquires a partially positive net charge. This transient positive ion state, formed by hot-hole transfer, dissipates to vibrationally excited states along a non-dissociative potential energy surface, leading to dissociation (Figure 7c). Essentially, both charge carriers, hot-hole, and hot-electron, concurrently contribute to the dissociation of chemisorbed O2 molecules with MOs strongly hybridized with the Ag(110) surface (Figure 7b).

Zhang et al. reveal a distinct mechanism that plasmonic heating can directly cause the expansion of nanoelectrodes, lead to the nanoswitching phenomenon [93]. In their experiment, MCBJ technique is used to stretch a metal wire, creating the metal molecular junction between to electrodes (Figure 7d). By applying light, the conductance switching behavior is stably reproduced between 1G_0_ and 80G_0_ (Figure 7e). Besides, dark field scattering spectra is measured under different samples. When approaching the plasmonic scattering peak, conductance start switching (Figure 7f), showing that the conductance change is related to the expansion of the electrodes due to plasmonic heating.

These insights offer a deeper understanding of the interplay among molecule junctions and LSP, paving the way for designing and controlling plasmon-induced reactions.

### 3.4 Conduction channels replacement under illumination

Recent research has revealed that light exposure can create new electron transmission channels in molecular junctions. A notable instance is the porphyrin-C_60_ dyad molecule, a combination of a porphyrin chromophore and a C_60_ electron acceptor [94]. Experiments employing the STM-BJ technique on indium tin oxide (ITO)-gold junctions unveiled that this molecule attains a charge-separated state upon illumination with a 520 nm laser [95].

Observations indicated a proportional increase in the fraction of molecules in a high conductance state, correlating linearly with laser power density, reaching up to 50 % at 200 mW/cm^2^, the threshold for potential photodamage. Transient absorption spectra revealed a durable charge-separated state of the dyad molecule on the ITO surface, distinct from its behavior in solution or within porphyrin films. Light absorption initiates photo-induced electron transfer, inducing a distinct state and prompting charge migration. This migration pathway involves either hopping to adjacent molecules or into the conductive ITO substrate. While the specific nature of the charge states remains elusive, the ITO substrate appears pivotal in extracting charge from the photoexcited state.

### 3.5 Molecule exciton-binding

**Figure 8:**
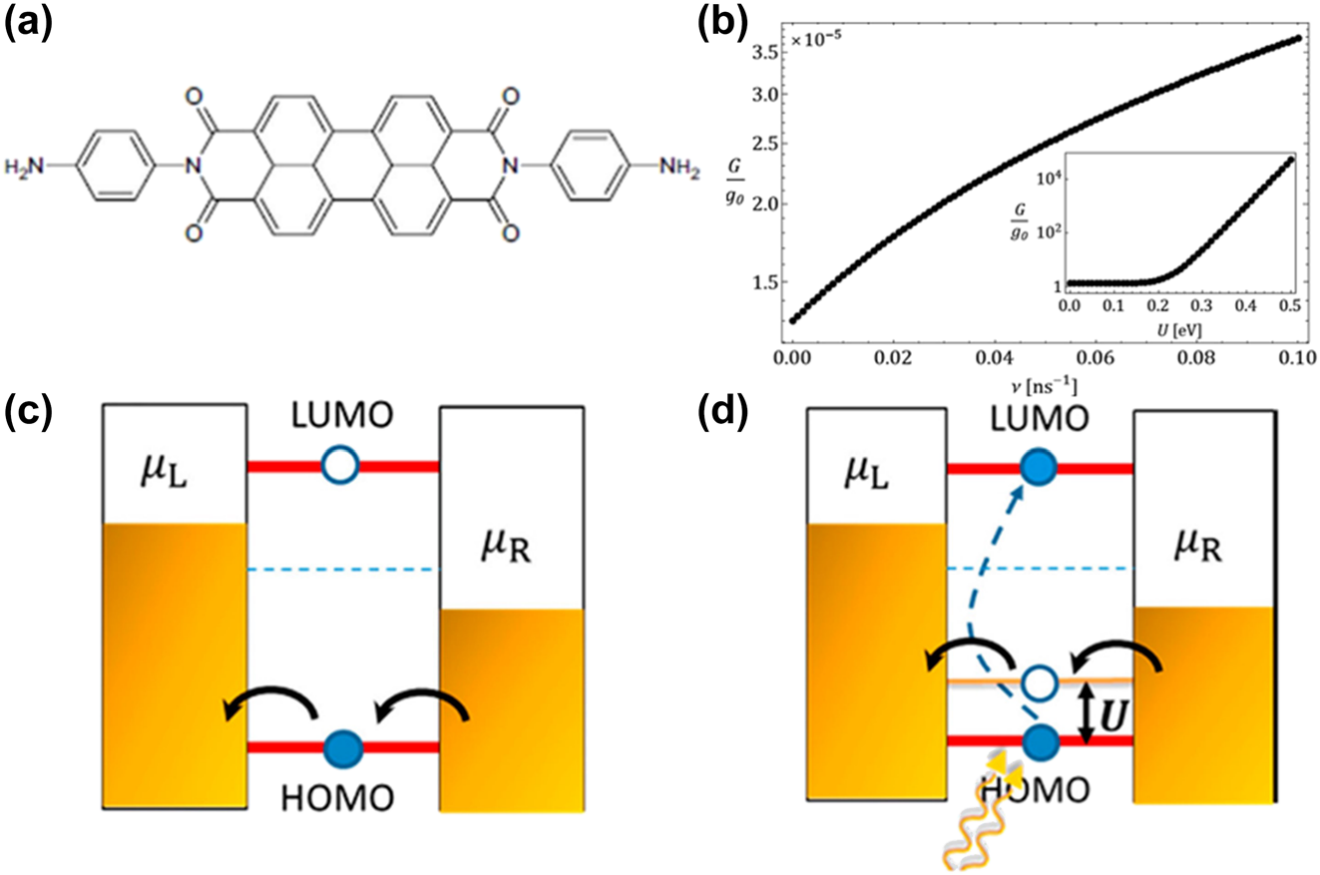
Structure and energy model of PTCDI molecule junction. (a) Schematic of the NH2-PTCDI-NH2 molecule structure. (b) Conductance as a function of the photoinduced HOMO–LUMO excitation rate *v*. Inset: conductance at a finite *v* as a function of the exciton binding energy *U*. (c, d) Schematic of band diagram shows that under dark, the current is dominated by hole transport through the HOMO. Under illumination, LUMO is partially filled and hole entered the HOMO, hence lifting the HOMO level toward the Fermi level to increase conductance. Figure (a–d) reprinted with permission from Ref. [96].

Zhou et al. recently demonstrated the manifestation of exciton-binding effects in symmetric single molecular junctions when illuminated by a laser, manifesting as the creation of an electron-hole pair within a molecular bridge [96]. Employing the STM-BJ technique, they immobilized an NH2-perylene tetracarboxylic diimide (PTCDI) –NH2 molecule between two gold electrodes via Au-amine bonds (Figure 8a). The conductance of PTCDI molecules exhibited significant and reversible alterations between measurements conducted in darkness and under 495 nm laser illumination. Notably, the laser’s energy aligned with the HOMO-LUMO gap of the PTCDI molecule (Figure 8b). In this specific junction configuration, the probable transport mechanism involves electron excitation from the HOMO to the LUMO upon resonant illumination, initiating intramolecular Coulomb interactions between electrons and holes. This process effectively shifts the HOMO level of the molecule closer to the Fermi level, thereby augmenting junction conductance (Figure 8c and d). This revelation opens a promising pathway for refining the design and performance of future molecular switches.

## 4 Molecular photo switches based on different single molecular junctions

Ensuring the consistent alteration of a single molecule’s conductance across multiple distinct states holds pivotal importance in advancing molecules for future optoelectronics, computing, and chemical or bio-sensing applications. Molecular junctions (MJs) have demonstrated conductance switching in response to diverse external stimuli such as electric fields [97], [98], [99], optic methods [100], chemical reactions and mechanical modulation [101]. Within this context, our focus centers on the conductance switching behavior in molecular junctions, particularly under the influence of optical excitation.

### 4.1 Azobenzene single-molecule junction

Among various photochromic materials, azobenzene derivatives hold a prominent position in the construction of photoswitching molecular junctions due to their wide availability and chemical resilience [102], [103], [104], [105]. Azobenzene, characterized by two phenyl rings linked by an N=N double bond, exhibits reversible switching between trans and cis conformations upon light exposure [106], [107], [108], [109].

In its trans configuration, azobenzene is nearly planar, while the cis conformation is more bent, with the phenyl rings twisted approximately 55° from each other. Ultraviolet (UV) light triggers the trans-to-cis isomerization, coinciding with the energy gap of the *π*–π* transition. Conversely, visible light prompts the cis-to-trans conversion, aligning with the *n*–π* transition [110], [111]. Typically, thermal energy can induce the cis-to-trans reversal in most azobenzene derivatives, rendering the trans isomer more stable at room temperature, although certain exceptions to this trend [112], [113].

#### 4.1.1 Metal-azobenzene derivative-metal single molecule junctions

**Figure 9:**
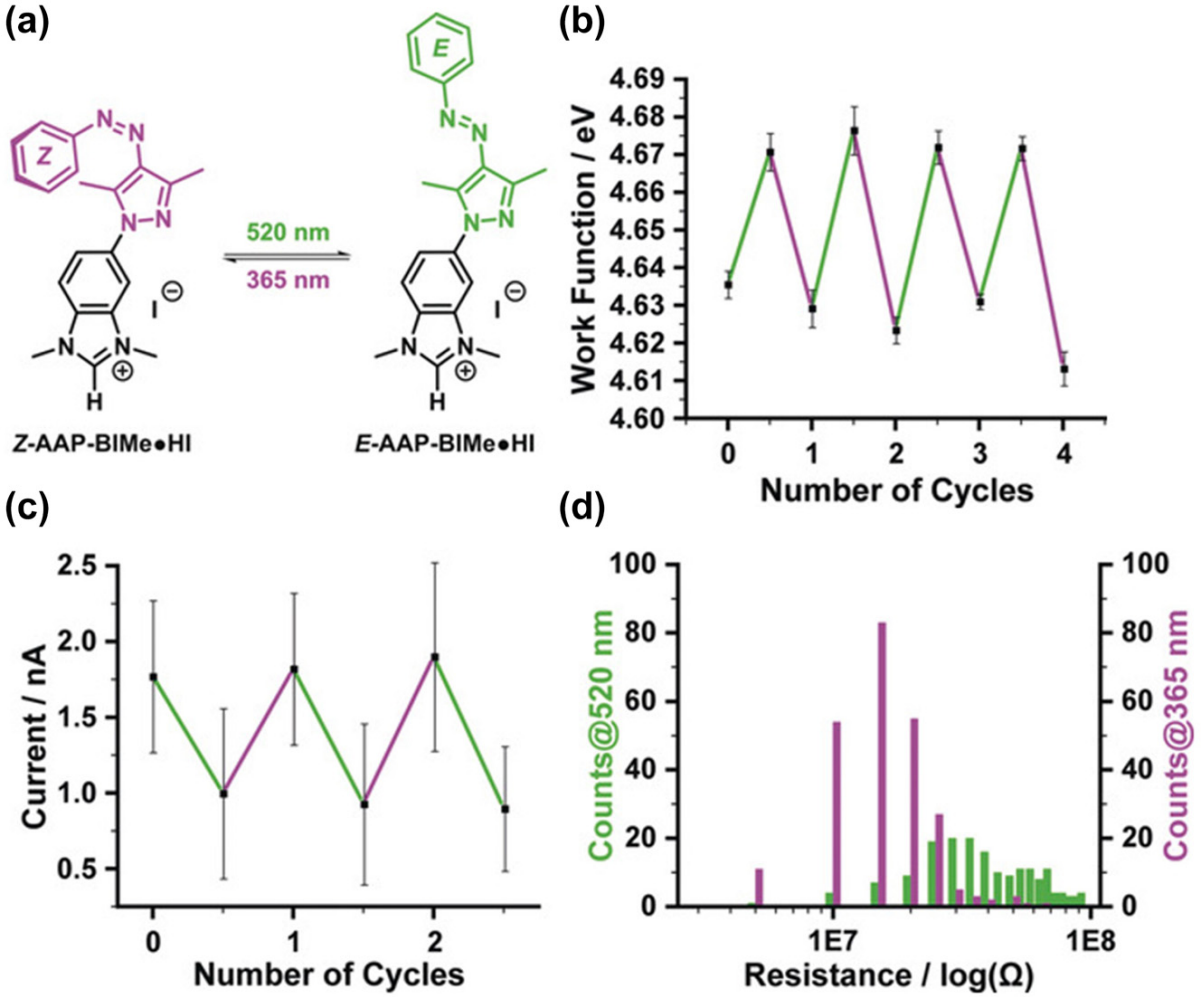
AAP-BIMe system photoisomerization measurement. (a) Schematic of AAP-BIMe⋅HI E/Z photoisomerization. (b) Conductance traces of cis and trans isomers of AzoTM single-molecule junctions. (c) Experimental symmetrized IETS spectra for cis and trans isomers at temperature *T* = 4.2 K. (d) Schematic of graphene-azobenzene-graphene junction and the structures of trans and cis isomers of azobenzene. Figure (a–d) reprinted with permission from Ref. [116].

Investigating the charge transport characteristics of azobenzene-based junctions has been a focal point in numerous studies targeting applications in photoswitching [56], [114], [115]. Nguyen et al. explored the conductance properties employing a unique azoheteroarene system, involving an arylazopyrazole (AAP) conjugated with an *N*-heterocyclic carbene (NHC), as illustrated in Figure 9a [116]. Selected for its exceptional photophysical attributes, AAP exhibited efficient photoisomerization in both forward and reverse directions. The NHC component facilitated anchoring of the molecule between gold electrodes, marking the first instance of a photoswitchable NHC being attached to a gold surface (Figure 9c). This attachment not only ensured stability but also effectively lowered the work function of the gold surface (Figure 9b). It was observed that the cis isomer of this system demonstrated a conductance 2.3 times higher than that of its trans counterpart. An alternative method to determine the state of azobenzene-based molecular switches (Figure 9d).

#### 4.1.2 Graphene-azobenzene-graphene single molecule junctions

**Figure 10:**
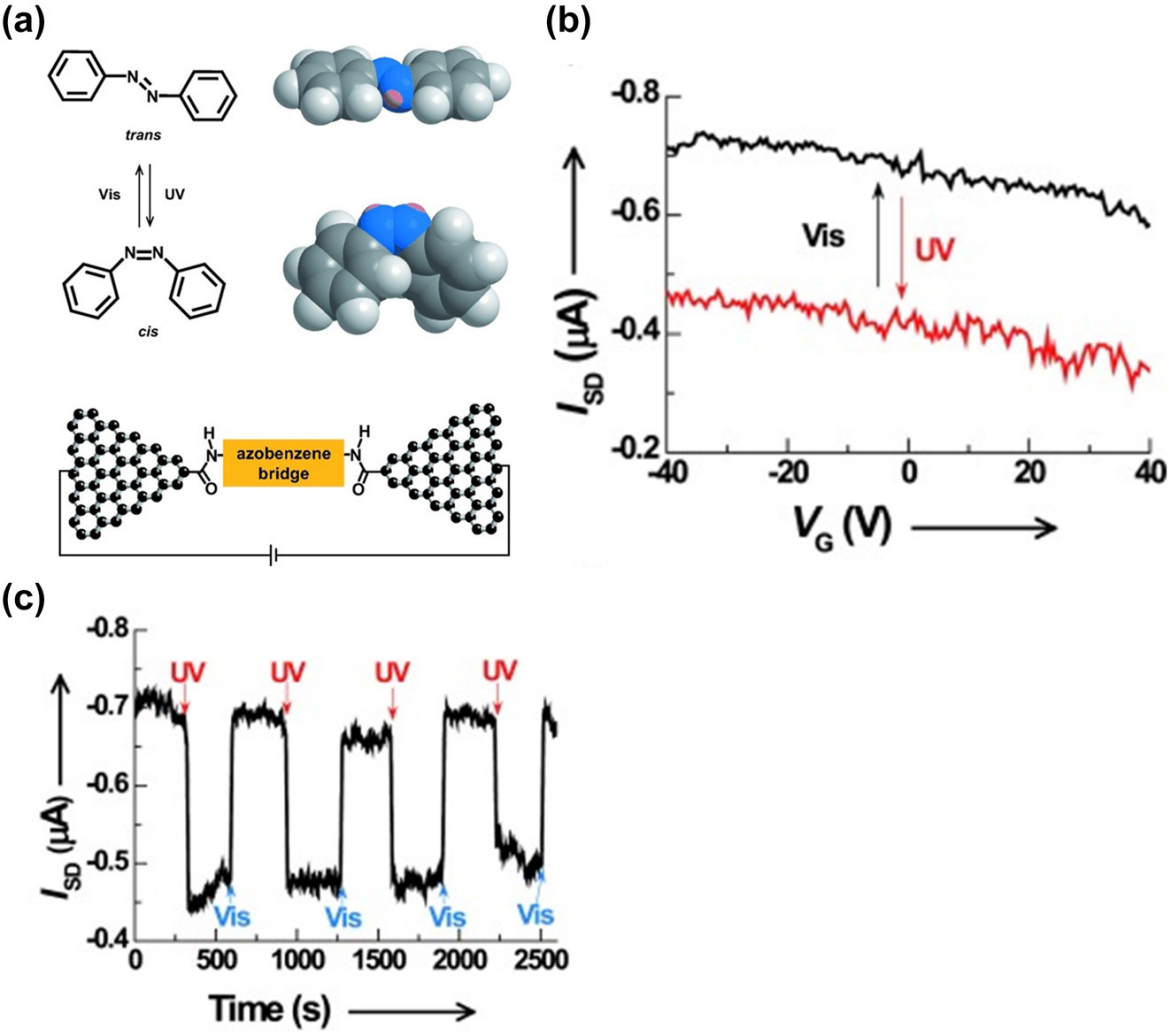
Azobenzene photoswitching measurement. (a) Schematic of graphene–azobenzene–graphene junction and the structures of trans and cis isomers of azobenzene. (b) Schematic of the *I*–*V* characteristics for the molecule responding to UV and visible light. *V*_SD_ = −50 mV. (c) Time trace of the drain current for the same device showing the reversible photoswitching events under irradiation with UV light and visible light. VSD = −50 mV; VG = 0 V. Figure (a–c) reprinted with permission from Refs. [102], [117].

Initially, metal–azobenzene–metal molecular junctions were synthesized ex-situ before integration into nanogapped electrodes [8], [117]. However, these molecular devices can become complex, as some previously used dithiolated molecules tend to oxidatively oligomerize and aggregate [118], [119]. Addressing these challenges necessitated the exploration of novel molecular structures beyond dithiolated varieties. Notably, Cao et al. engineered an azobenzene molecule equipped with terminal amino groups [102]. These amino groups, known for their stability, facilitate the attachment of azobenzene to graphene nanogap electrodes through strong covalent amide bonds, enhancing the durability and reliability of graphene–azobenzene–graphene single-molecule junctions (Figure 10a) [120], [121]. The study examined how junctions with an azobenzene bridge reacted to different light wavelengths. Under UV illumination (254 nm), the azobenzene unit switched from trans to cis conformation, leading to reduced conductance due to a wider HOMO-LUMO energy gap in the cis isomer. The original conductance levels were nearly restored by reversing the photoisomerization using visible light. To assess the stability and reversibility of the switching, they performed multiple UV and visible light irradiation cycles, tracking the current changes in real time under constant voltage conditions (Figure 10b). The graphene–azobenzene–graphene junction demonstrated stable and reversible photoswitching phenomenon between two distinct conductive states even after long time numerous cycles (Figure 10c). This breakthrough marks the debut of a reversible light-activated single-molecule switch, promising significant potential for diverse functional molecular electronic devices in practical applications.

### 4.2 Dihydropyrene single molecule junctions

**Figure 11:**
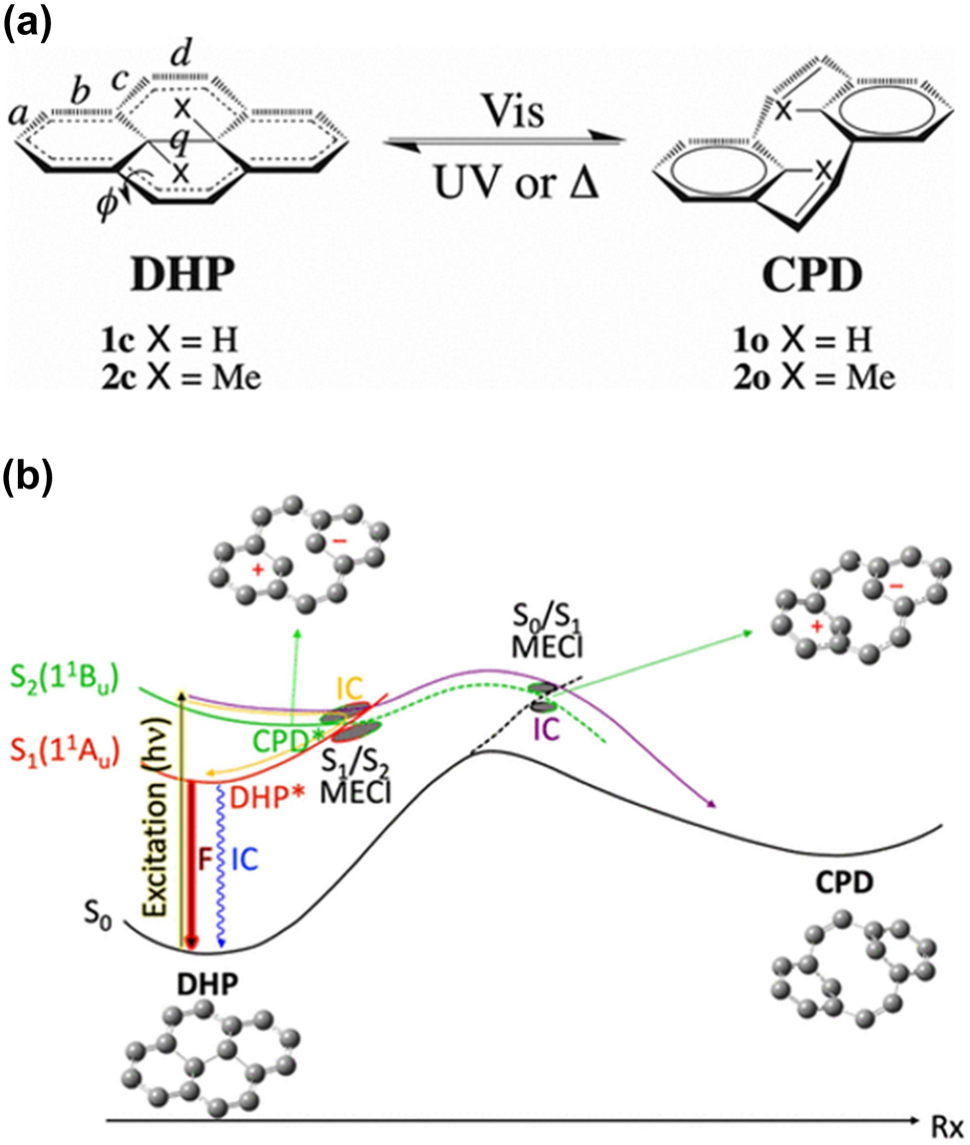
Structure and energy model of DHP/CPD system. (a) Schematic of the DHP/CPD photochromic couple. Labels used for geometrical parameters. (b) Schematic of potential energy profiles of the relevant electronic states involved in the DHP/CPD system. Figure (b) reprinted with permission from Ref. [128].

Dihydropyrene and cyclophanediene (DHP and CPD) represent notable photochromic isomers, depicted in Figure 11a. DHP, renowned for its expansive *π*-conjugated planar structure, undergoes a conversion to the less *π*-conjugated CPD isomer upon exposure to visible light [122], [123], [124]. Typically, colorless and open, the CPD form can reversibly transform back into the colored, closed DHP form through UV illumination or heat application [125], [126]. This unique DHP/CPD system exemplifies a negative photochrome, where a colored, thermodynamically stable form shifts to a decolored form Ref. [127].

The intricate polycyclic hydrocarbon framework of DHP offers versatility in chemical functionalization, rendering it an attractive prospect for developing intelligent optoelectronic materials and molecular devices [129], [130], [131]. In the recent research from Lognon et al., spin-flip time-dependent density functional theory (SF-TD-DFT) is used to go into the photoisomerization mechanism of the DHP photochromic system [128]. By study the conical intersection to identify the different electronic states, they observed the crossings between covalent and ionic states (Figure 11b). The switching process within the DHP–CPD system involves considerably lesser conformational reorganization compared to other photochromic molecules. Nevertheless, the achievement of efficient switching in a purely solid-state form remains an ongoing challenge for this specific system.

### 4.3 Diarylethene single molecule junctions

Diarylethene (DAE) derivatives represent another prominent category of photochromic compounds. These compounds, initially colorless and in a ring-open form, undergo electrocyclization upon UV light exposure to create a ring-closed isomer [132], [133], [134], [135]. This closed form can revert to the original open structure when exposed to visible light. The closed isomer of DAE is nearly planar and conjugated, allowing for delocalization of *π* electrons across the molecule. In contrast, the open form is nonconjugated and adopts a bent structure, with the thiophene rings twisted away from the cyclopentene ring, restricting *π* electron delocalization to each half of the molecule and interrupting electronic communication through the central ring. Consequently, the two isomers exhibit different conductive states [135], [136]. Diarylethene molecules have been extensively studied for optical switching applications due to their exceptional attributes, including fatigue resistance, thermal stability of both isomers, rapid response times, high reversibility of photoisomerization, and high quantum yields [137], [138], [139], [140], [141]. Furthermore, unlike azobenzene derivatives, diarylethene molecules experience negligible changes in molecular length during photoisomerization [100], [142]. As a result, diarylethene derivatives show significant promise for use in stable photoswitching molecular devices [5], [143], [144].

#### 4.3.1 Metal-diarylethene-metal single molecule junctions

**Figure 12:**
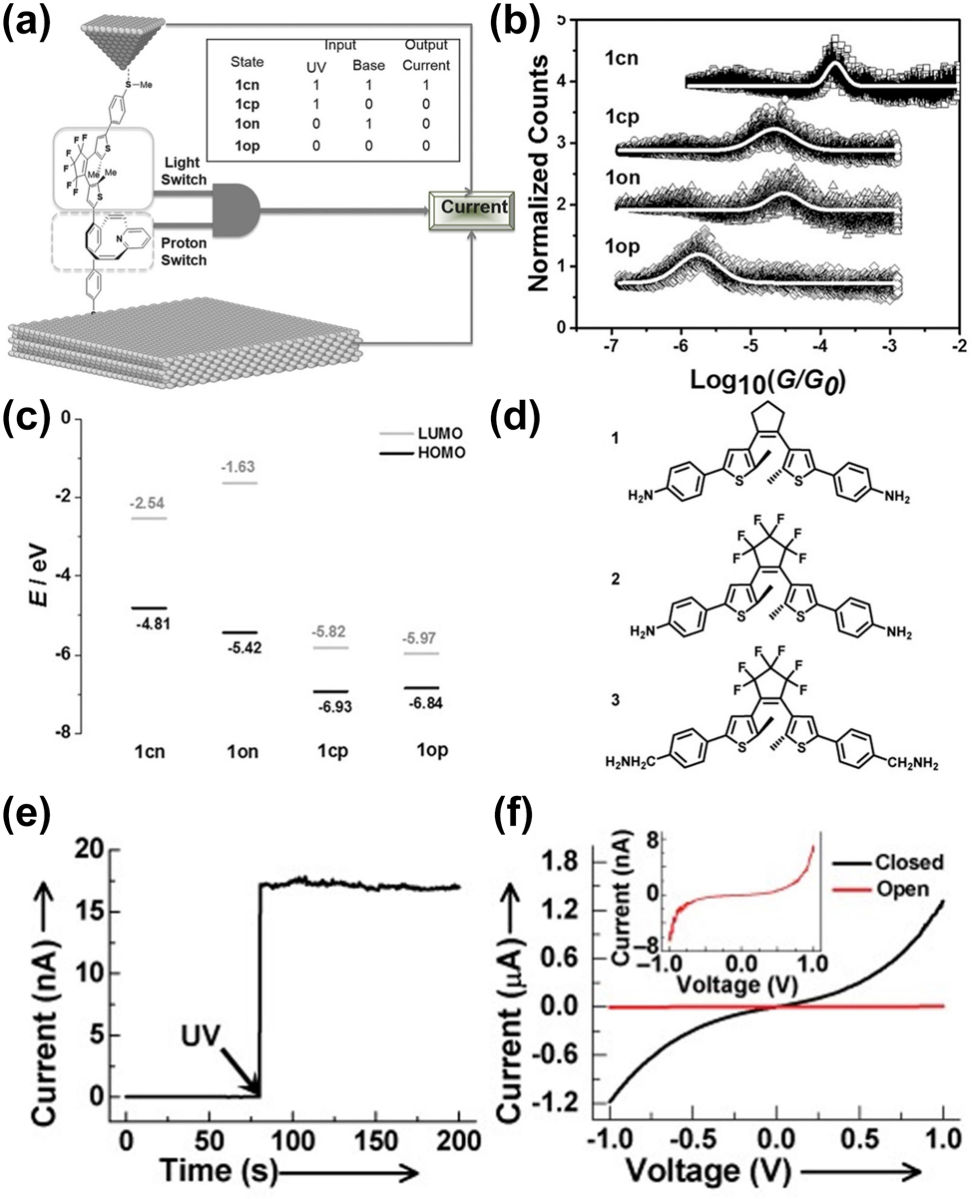
SAM AND logic gate device and DAE photoswitching experiment. (a) Illustration of the SAM AND logic gate molecules in between the Au tip and substrates. Inset: truth table of four states. (b) Conductance histograms of 1cn, 1cp, 1on, 1op states. The conductance peaks are fitted using Gaussian function. (c) Energy-levels diagram for 1cp, 1on, and 1op constructed from DFT. (d) Schematic of DAE 1–3 structure. (e) The current-time, curve of the same device at *V*_D_ = 50 mV and *V*_G_ = 0 V. (f) *I*–*V* characteristics of DAE molecular junction with open and closed states at *V*_G_ = 0 V. Inset: The enlarged *I*–*V* curve for the open state. Figure (a–f) reprinted with permission from Refs. [100], [145].

Zhang et al. utilized the STM-BJ technique to create a single-molecule logic gate [145]. This gate combined a light-switchable diarylethene (DAE) unit and a proton-switchable edge-on gated pyridinoparacyclophane (PPC) unit (Figure 12a). To prevent quenching of the DAE’s excited state, the PPC unit was positioned near the gold substrate, and a thiomethyl group was added to the DAE end. The design aimed to minimize quenching of the DAE’s excited state by positioning the PPC unit near the gold substrate and introducing a thiomethyl group at the DAE end, effectively segregating the orbital mixing of PPC and DAE components and allowing for independent switching of the two units [146].

Resulting from this design, the single-molecule system exhibited four discernible states: 1op (open DAE, protonated PPC), 1on (open DAE, neutral PPC), 1cp (closed DAE, protonated PPC), and 1cn (closed DAE, neutral PPC). The conductance measurements for these states in single-molecule junctions showed significant variations (1op: 1.7 × 10^−6^ G_0_, 1on: 3.0 × 10^−5^ G_0_, 1cp: 2.2 × 10^−5^ G_0_, 1cn: 1.6 × 10^−4^ G_0_) as depicted in Figure 12b. The 1cn state, achieved under UV light in the neutral state, exhibited the highest conductance due to its extended conjugation and alignment of its HOMO with the Au Fermi level. In contrast, the 1op state displayed the lowest conductance, as its conjugation was disrupted, and its frontier orbital (LUMO) was distant from the Au Fermi level (Figure 12c). This research presents a novel approach in constructing intricate single-molecule electronic devices by integrating two interdependent functional units.

#### 4.3.2 Graphene-diarylethene-graphene single molecule junctions

In their study, Jia et al. delved into conductance switching and charge transport mechanisms within single-molecule junctions based on diarylethene (DAE) [100]. Their methodology involved immobilizing DAE molecules onto nano-gapped graphene electrodes using robust covalent amide bonds, ensuring the junction’s resilience to various external stimuli [120], [121]. To fine-tune the energy level alignments at the molecule/electrode interface, they modified the DAE backbones with specifically chosen groups (Figure 12d). This modification involved two key changes: replacing the hydrogenated cyclopentene in DAE 1 with a fluorinated unit in DAE 2 to decrease electron density on the central alkene unit and enhance fatigue resistance and introducing CH2 between the functional center and terminal amine group in DAE 3, disrupting *π*-electron delocalization and reducing electronic interaction with the electrodes [147].

Under UV light exposure, DAE molecules transitioned from an open, nonconjugated isomer to a closed, conjugated form, prompting a shift in single-molecule junctions from a low-conductance (off) state to a high-conductance (on) state (Figure 12e) [148], [149]. Remarkably, the on/off conductance ratio progressively increased across the DAE variants, from 60 in DAE 1 to 200 in DAE 2 and up to 300 in DAE 3 (Figure 12f). The zero-bias voltage transmission spectra indicated that the conductance switching was a result of changes in molecular energy levels due to configuration transformation or side group substitution [150], [151]. Further, both transition voltage spectroscopy (TVS) and first-principles calculations confirmed the ability to modulate the molecule-electrode coupling strength through molecular engineering. This modulation led to a photo-gated inflection transition in the charge transport mechanism from direct to Fowler–Nordheim tunneling [152], [153], [154]. These findings offer valuable insights into the design of new molecule-based devices and the interplay between the electronic structures of molecular junctions and their charge transport mechanism.

**Figure 13:**
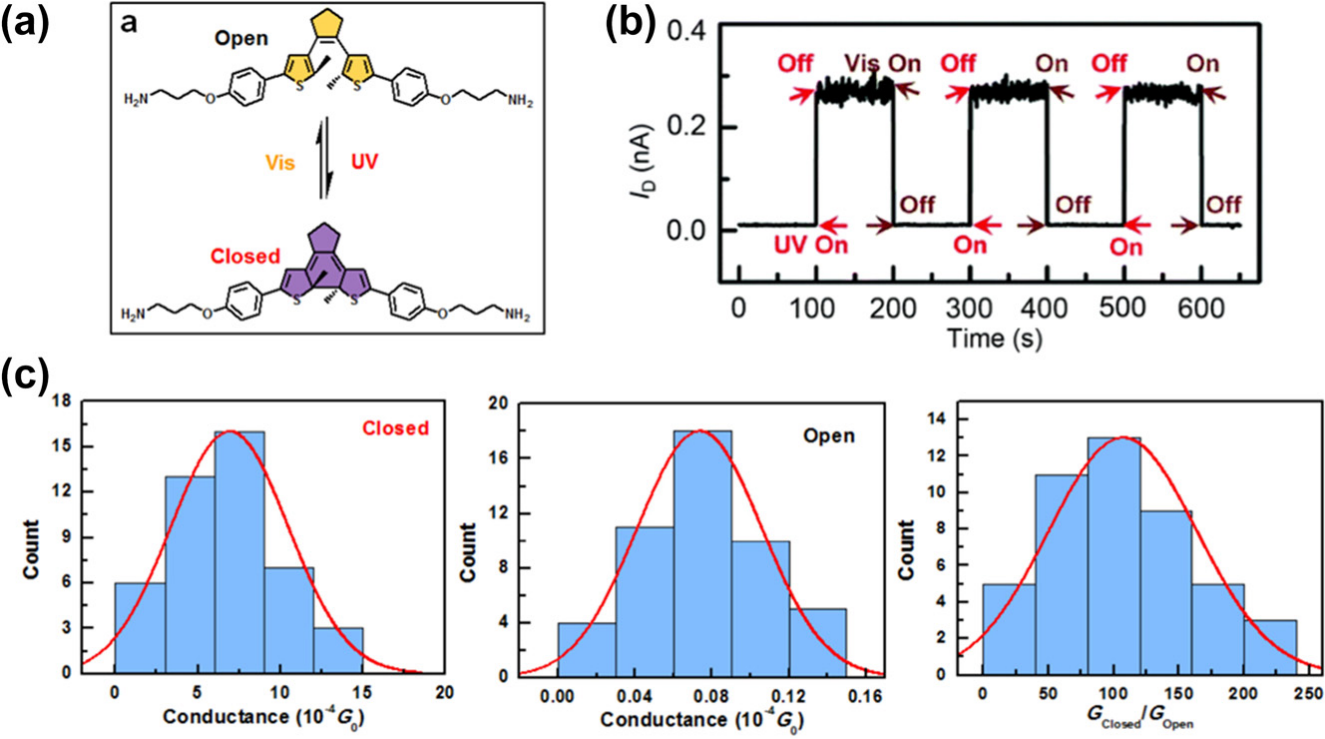
Illustration of DAE photoswitching measurement. (a) Schematic of graphene-diarylethene-graphene junction that highlights the expansion of the molecular bridge by methylene groups. (b) Real-time measurement of the current through a diarylethene molecule that reversibly switches between the closed and open forms under UV and visible radiation. *V*_D_ = 100 mV and *V*_G_ = 0 V. (c) Conductance histogram of the diarylethenes molecule junctions in the closed state (left), open state (middle) and the conductance ratio (right). Figure (a–c) reprinted with permission from Ref. [5].

In the further study by Jia et al., when DAE molecules in closed form were sandwiched between graphene electrodes and irradiated with UV light, they did not revert to the insulating open form. This was attributed to energy transfer from the photoexcited DAE molecule to the carbon electrodes’ extended *π*-electron system [100], [149]. Conversely, in gold-DAE-gold single-molecule junctions, a unidirectional photoswitching phenomenon occurred, linked to the quenching of excited states in open isomers near Au electrodes [148]. This indicates the significant influence of molecule-electrode coupling strength on device performance. To mitigate this quenching effect, DAE molecules were modified by adding three CH2 groups on each side of the molecular backbone (Figure 13a) [5]. This modification resulted in narrow resonance half-widths of transmission peaks, signifying a weaker graphene/molecule interfacial coupling around 1 meV for both isomers [100]. Theoretical analysis revealed that the DAE molecules’ HOMO was the only orbital near the graphene electrodes’ chemical potentials, allowing conduction within a −1 V to 1 V bias range. Notably, the HOMO’s offset from the graphene electrodes’ Fermi level was larger for the open conformation than for the closed one. The real-time current measurement of the graphene–diarylethene–graphene junctions showed that they could switch reversibly between on and off states upon exposure to UV and visible light (Figure 13b). This switching was robust and reproducible over more than 100 cycles at room temperature. It shows with an on/off ratio of about 100, with high conductance of 6.94 ± 3.52 × 10^−4^ G_0_ and low conductance of 7.46 ± 3.33 × 10^−6^ G_0_ (Figure 13c). Furthermore, Guo et al. investigated the temperature-dependent charge transport mechanism in these junctions [5], [155]. They found that above 90 K, the torsional vibration of the molecule’s phenyl rings was thermally activated, increasing vibronic coupling and creating additional conductance channels. Consequently, charge transport exhibited a transition from coherent tunneling at low temperatures to incoherent transport at higher temperatures [156]. This incoherent transport had different activation energies due to the electron/phonon coupling effect, varying with the molecular energy levels of the two conformations. Thus, the charge transport mechanism in these junctions was influenced by both temperature and molecular energy levels.

In addition to the previously discussed photochromic molecules, other molecules exhibit comparable photochromic behaviors. For example, dihydroazulene/vinylheptafulvene photochromic system can reversibly switches between two distinct geometric and conductive states, showing higher quantum yields compared to azobenzenes [157], [158]. Moreover, norbornadiene-quadricyclane isomers and dihydrodibenzo[a,c]phenazine derivatives have been investigated for exploring photo-induced charge transport mechanisms in single MJs [159], [160].

## 5 Photo-induced electron transport in self-assembled monolayers (SAM) of single molecule junctions

Beyond single molecule junction investigations, SAM junctions, Research into SAM (self-assembled monolayer) junctions extends beyond single molecule junction investigations. SAM junctions, where multiple molecules are interconnected in parallel, present a significant domain within optoelectronic research. While single-molecule break junction investigations usually entail examining conductance versus displacement traces, the exploration of SAM junctions predominantly revolves around assessing current density concerning junction bias to elucidate their transport characteristics.

### 5.1 Photo switches build by different SAM molecule junctions

**Figure 14:**
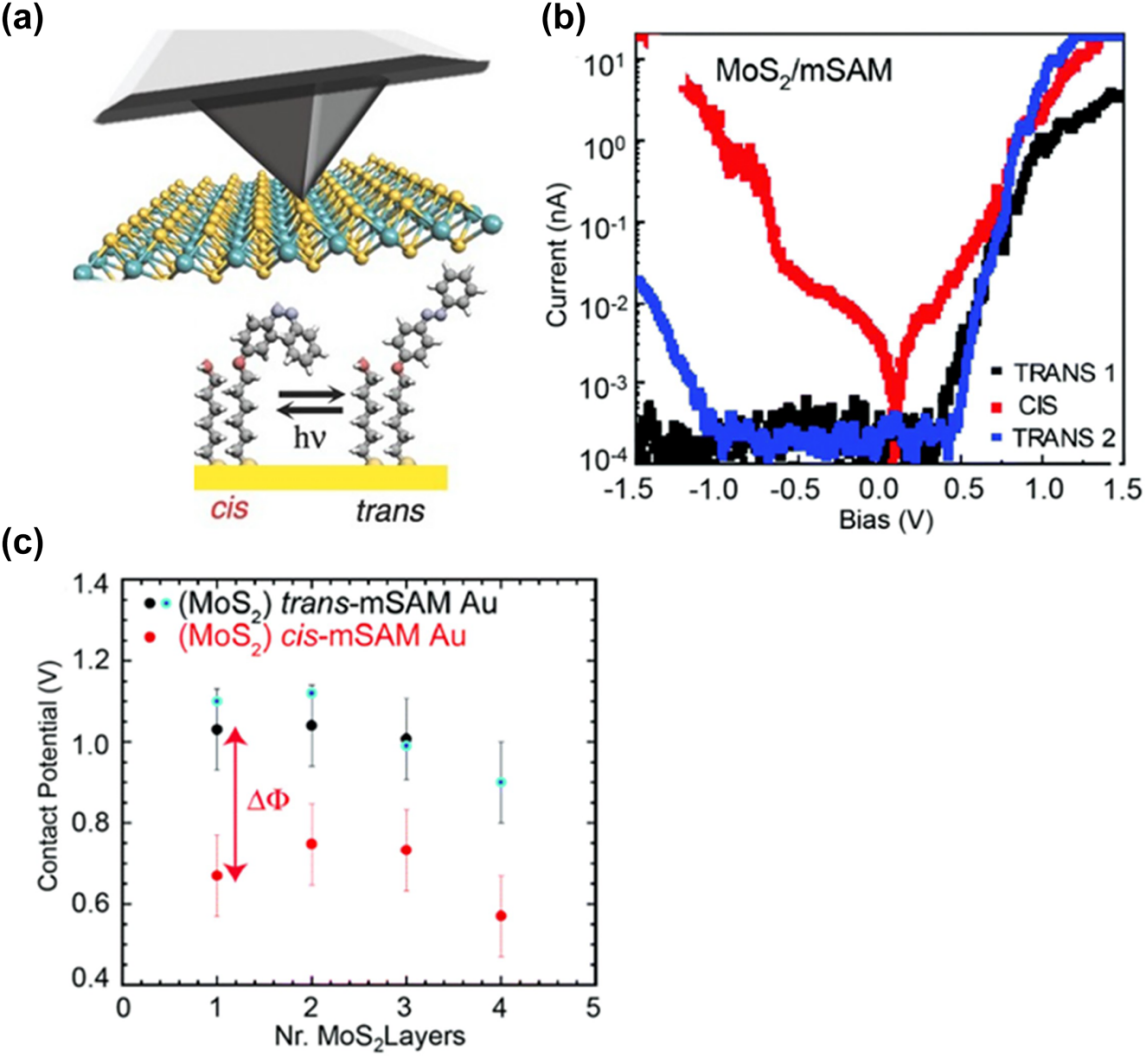
Azobenzene derivatives SAM photoswitching device. (a) Schematic of the Au-azobenzene derivatives junction measurement. (b) *I*–*V* characteristics in a semilogarithmic scale for the MoS2-mixed-SAM junction before (trans1), after UV (cis), and after white light exposure (trans2). (c) KPFM results recorded from different layers of MoS2 on mSAM-Au using a Pt–Ir tip as the probe. Figure (a–c) reprinted with permission from Ref. [73].

In exploring the photoswitching properties of various photochromic molecules within SAM (self-assembled monolayer) junction configurations, a range of studies has been conducted, particularly focusing on azobenzene, diarylethene, and dithienylethene [144], [161], [162], [163], [164], [165], [166]. Margapoti et al. have extensively studied AzoC6, a photoisomerizable azobenzene derivative, and used it to create a photoswitchable molecular junction (Figure 14a) [73]. To prevent aggregation that could impede photo-mediated molecular conformational switching [167], they designed a mixed self-assembled monolayer (mSAM) of AzoC6 and 6-(2-mercapto)-1-hexanol molecules in a 1:1 ratio. This mSAM was layered on an Au substrate, with varying layer thicknesses of MoS2 exfoliated on top, establishing an Au-mSAM-MoS2-Pt-Ir probe junction [168], [169], [170]. This molecular device exhibited both photoswitching and rectifying characteristics (Figure 14b). Under UV-light (366 nm) irradiation, the 1L-MoS2-based molecule junction experienced a conformational change of AzoC6 molecules from trans to cis, causing a nearly 1.5 magnitude order increase in current density in the forward bias and a complete suppression of rectification. The rectifying property was restored upon overnight white light irradiation. Charge transport through these Au-mSAM-MoS2-Pt-Ir probe junctions, considering the comparably long HS-C_10_H_21_ molecule, was attributed to a combination of tunneling through a metal-semiconductor (MS) barrier and the mSAM layer [171]. The photoswitchable transport features were deduced from significant differences in the contact potentials of MoS2-trans-mSAM and MoS2-cis-mSAM (Figure 14c). The rectification mechanism was linked to the misalignment of the conduction band of the trans-mSAM with the Fermi level of the Au electrode.

**Figure 15:**
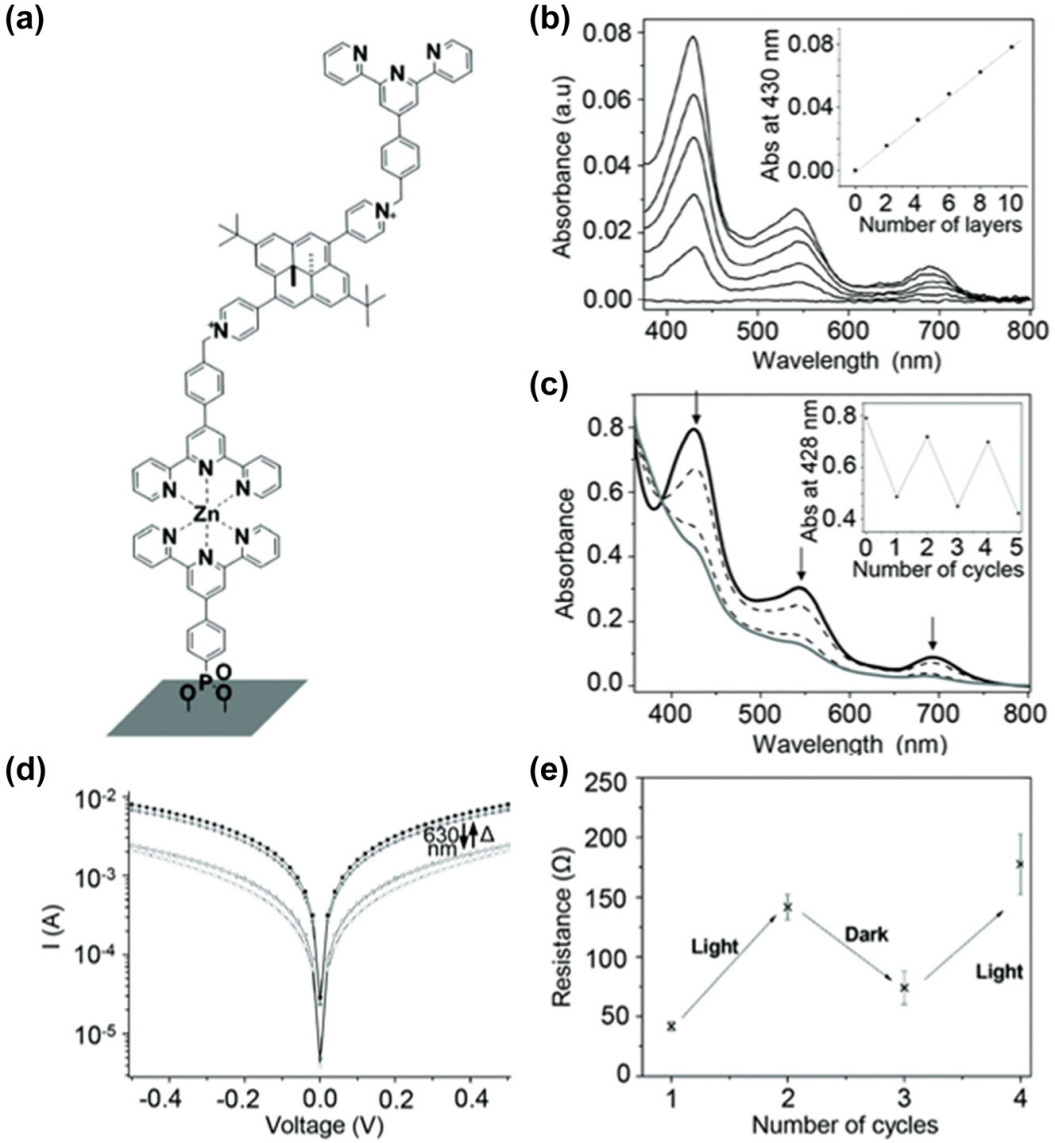
DHP photoswitching experiments on ITO surface. (a) Structure of ITO/Zn-tpy-DHP-tpy film on the ITO surface. (b) UV-visible absorption spectra of ITO/(Zn–tpy–DHP–tpy)_*n*_ recorded during the construction of successive assemblies from *n* = 2 to 10 of Zn–tpy–DHP–tpy layers. Inset show absorbance at 430 nm versus number of layers. (c) Evolution of the UV-visible spectra of the ITO/(Zn–tpy–DHP–tpy)_10_ film recorded during irradiation (closed form: black line, open form: gray line). (d) *I*–*V* curves (semilog scale) of the ITO/(Zn–tpy–DHP–tpy)_5_/Ti multilayer before (full square) and after (empty square) red irradiation and after subsequent thermal relaxation to restart the original state. (e) Reverse of the conductance at +0.5 V for ITO/(Zn–tpy–DHP–tpy)_5_/Ti. Figure (a–e) reprinted with permission from Ref. [131].

Bakkar et al. introduced high-performance coordination polymers with a photochromic core for thin film applications (Figure 15a) [131]. These films were grown on indium tin oxide (ITO) surfaces by first preparing self-assembled monolayers (SAMs) of 4-(2,2′:6′,2″-terpyridine-4-yl)-phenylphosphonic acid on the ITO. Subsequent coordination reactions involved alternating dips of the ITO substrate into solutions of zinc metal ion and terpyridine (tpy)–DHP–tpy. The utilization of zinc ions prevented interference with the light absorption of the photochromic core, maintaining high photo-conversion efficiency [172]. The UV–vis absorption spectra during the layer-by-layer assembly indicated distinct absorption bands related to *π*–π* transitions in the DHP core, with absorbance linearly increasing with the number of layers (Figure 15b). Red light irradiation triggered a decrease in these absorption bands, suggesting exclusive isomerization in the ITO-(Zn–tpy–DHP–tpy)10 film (Figure 15c). Reversible bidirectional photoswitching was achieved in these junctions, with the closed (DHP isomer) form exhibiting higher conductivity than the open (CPD isomer) form (Figure 15d and e). The conductance decreased significantly after red light illumination, and the junctions maintained their electrical characteristics over multiple illumination cycles. This approach offered a promising platform for studying the electrical and optical properties of photochromic materials [125], [126], fostering the development of molecular devices.

**Figure 16:**
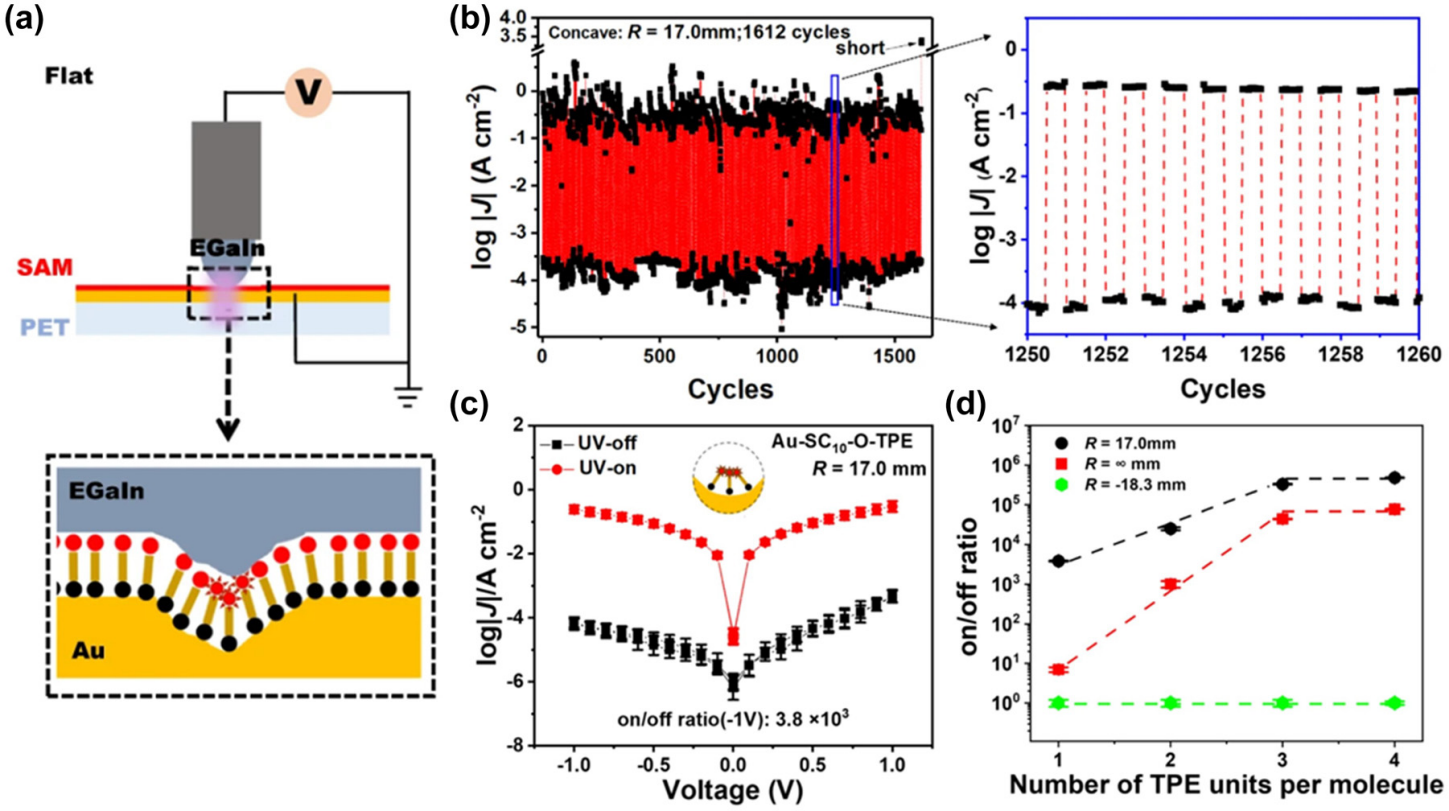
TPE SAM photoswitching device. (a) Schematic of flat PET/Au–SC_10_–O-TPE/Ga_2_O_3_/EGaIn junction with a UV lamp focused below the junction. (b) The real-time UV-on/off cycle and zoom-in image of sustained switching versus time over ten consecutive cycles with UV on and off. (c) The log|*J*|/(*V*) curves of the Au–SC_10_–O-TPE junction in the UV-off (black square) and on (red dot) state. *J* = current density. (d) The on/off ratio as a function of number of TPE units per molecule at three different bending geometries. Figure (a–d) reprinted with permission from Ref. [173].

Yang et al. successfully developed a method for fully reversible *in-situ* optoelectronic switching in SAMs of tetraphenylethylene (TPE) molecules [173]. By bending the supporting electrodes, they optimized aggregation-induced emission (AIE) in the SAMs (Figure 16a), achieving substantial and consistent on/off ratios (Figure 16c). The photoswitching process demonstrated full reversibility over more than 1600 cycles (Figure 16b). Moreover, synthesizing three additional AIE-active molecules, each linked to multiple TPE terminal groups, led to a marked exponential increase in the on/off ratio. The increase correlated with the count of TPE units in each molecule, reaching a peak ratio of approximately (4.8 ± 0.1) × 10^5 (Figure 16d). An enhancement in performance was observed by maximizing phenyl density with the bulky tetra-TPE design. This outcome underscores the mechano-optoelectronic response’s tailorability in these junctions. The ultra-high photoswitching efficiency achieved at maximal phenyl density emphasizes the devices’ potential for unique applications requiring integrated mechanical and photonic stimuli or responses.

### 5.2 Photo-induced transport in SAM molecule junctions

**Figure 17:**
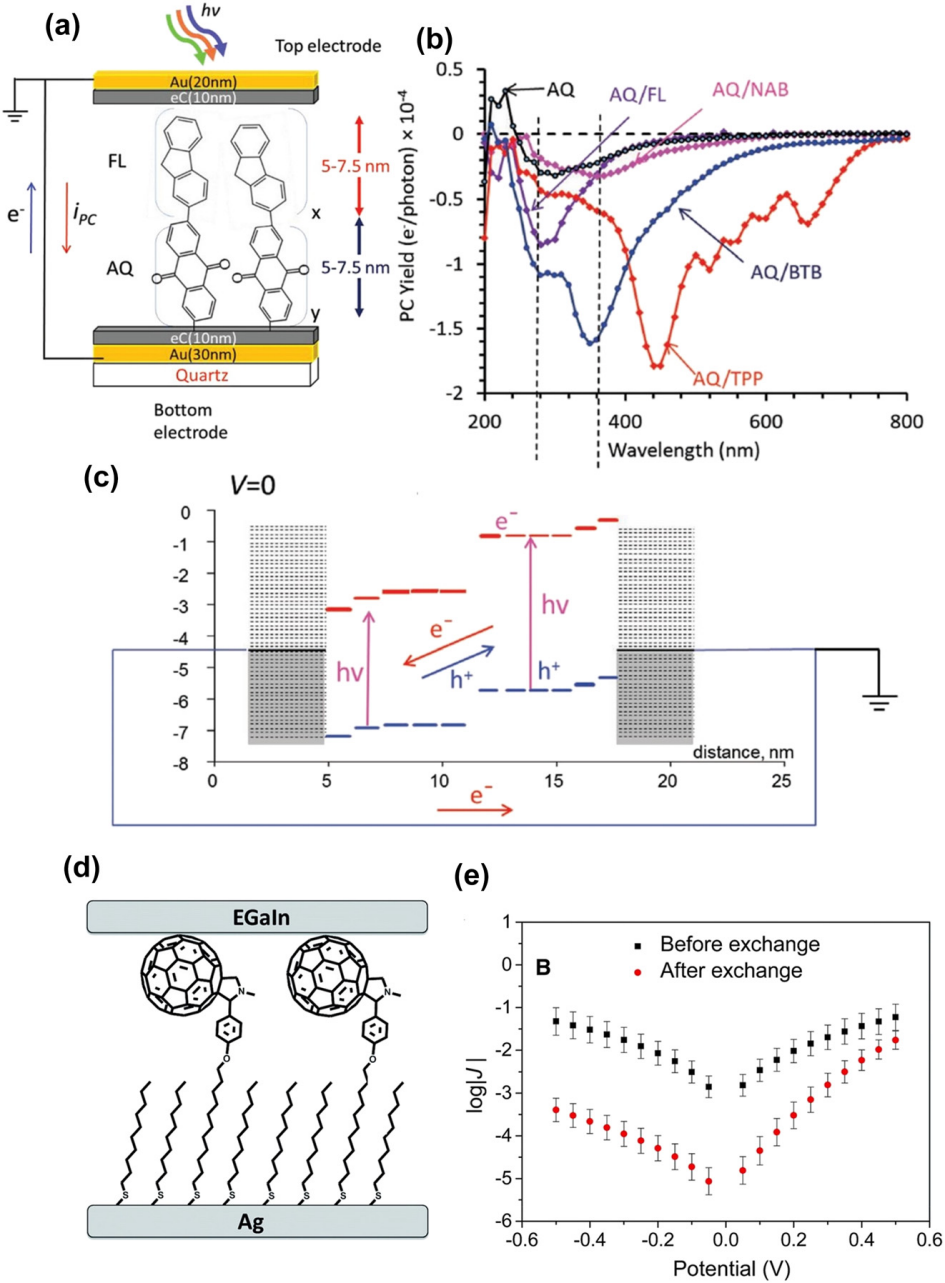
Charge transport properties and rectification behavior by investigating different SAM junctions. (a) Schematic of Au_30_/eC_10_/AQ_6_/FL_6_/eC_10_/Au_20_ bilayer MJ structure. (b) Photocurrent collected for four bilayers MJs having BTB as a first (bottom) layer and single-layer BTB as a reference. (c) Schematic mechanism for photocurrent production in an AQ/FL bilayer MJ at zero bias, with HOMO (blue) and LUMO (red). (d) Schematic of Ag^TS^/FSC11/Ga_2_O_3_/EGaIn SAM junction. (e) Plots of log|*J*| versus *V* for SAMs of SC10 on Ag_TS_ before and after incubation with FSC11. *J* = current density. Figure (a–e) reprinted with permission from Refs. [175], [178].

SAM junctions have emerged as promising elements for solid-state fabrication, offering potential applications in photo-detection, lighting, and energy harvesting. Recent investigations have delved into molecular species exhibiting light-tunable transport properties [174], [175], [176], [177]. In a study by Shailendra et al., Au-carbon-bilayer molecular junctions were explored, featuring distinct 5–7 nm thick molecular layers between carbon contacts [175]. The research aimed to elucidate how the molecular orbitals and optical absorbance spectra of these oligomers influence the photocurrent response, charge transport direction, and peak response wavelength (Figure 17a). The results revealed a close correspondence between the photocurrent spectrum and the absorption spectrum of the molecular layer, indicating electron-hole generation as the source of the photocurrent. Notably, the interface between two molecular layers, such as anthraquinone/fluorene and other combinations, primarily determined the photocurrent polarity, as depicted in Figure 17b. In an unbiased bilayer anthraquinone/fluorene molecular junction, an upward potential shift in the donor molecule’s HOMO and LUMO energies was observed, while the acceptor’s energies shifted downward. This electrode-molecule and organic/organic interface interaction induced an internal electric field that drove the photocurrent (Figure 17c). Under external bias, the photocurrent in the illuminated SAM junctions notably surpassed the dark current, exhibiting a multiplication factor ranging from 102 to 105, depending on the bias, bilayer structure, and wavelength. These findings position these junctions as promising photodetectors, achieving an internal quantum efficiency of 0.14 electrons per absorbed photon.

Qiu et al. demonstrated current rectification in molecular junctions employing SAMs of FSC11, a derivative of C_60_ functionalized with 11-unedecanethiol (SC11) (Figure 17d) [178]. Upon replacing SC11 with FSC11, log|*J*| decreased by approximately two orders of magnitude at negative bias, resulting in rectification (Figure 17e). Theoretical calculations revealed that the maximum rectification was correlated with the structure of the C_60_ cage, specifically, the localization of the lowest-unoccupied *π*-state (LUPS) to the C_60_ *π*-system in contact with and pinned to the Ga_2_O_3_/EGaIn electrode. Positive bias decreased the Fermi energy Ef at that electrode, subsequently reducing the LUPS and bringing it into resonance with Ef at the Ag^TS^ electrode. This alignment rendered the LUPS energetically accessible, facilitating charge tunneling from Ag^TS^ onto the C_60_ cage rather than from Ag^TS^ to Ga_2_O_3_/EGaIn.

## 6 Raman sensing in the molecular junctions

Achieving *in situ* observation of the geometric and structural kinetics and dynamics at the single molecule level has been a long-term objective in chemistry. It is also essential for comprehending the structure-property relationships and transport mechanisms of molecules in electronic devices. To delve deeper into the impact of the molecular core and its bonding to electrodes on electrical properties, researchers have employed advanced optical detection methods, like Raman spectroscopy. This technique is used to capture vibrational fingerprints of molecular junctions (MJs), allowing for the simultaneous acquisition of electrical and spectroscopic data. This innovative approach provides unique insights into the molecular-level mechanisms across a broad range of molecular species.

**Figure 18:**
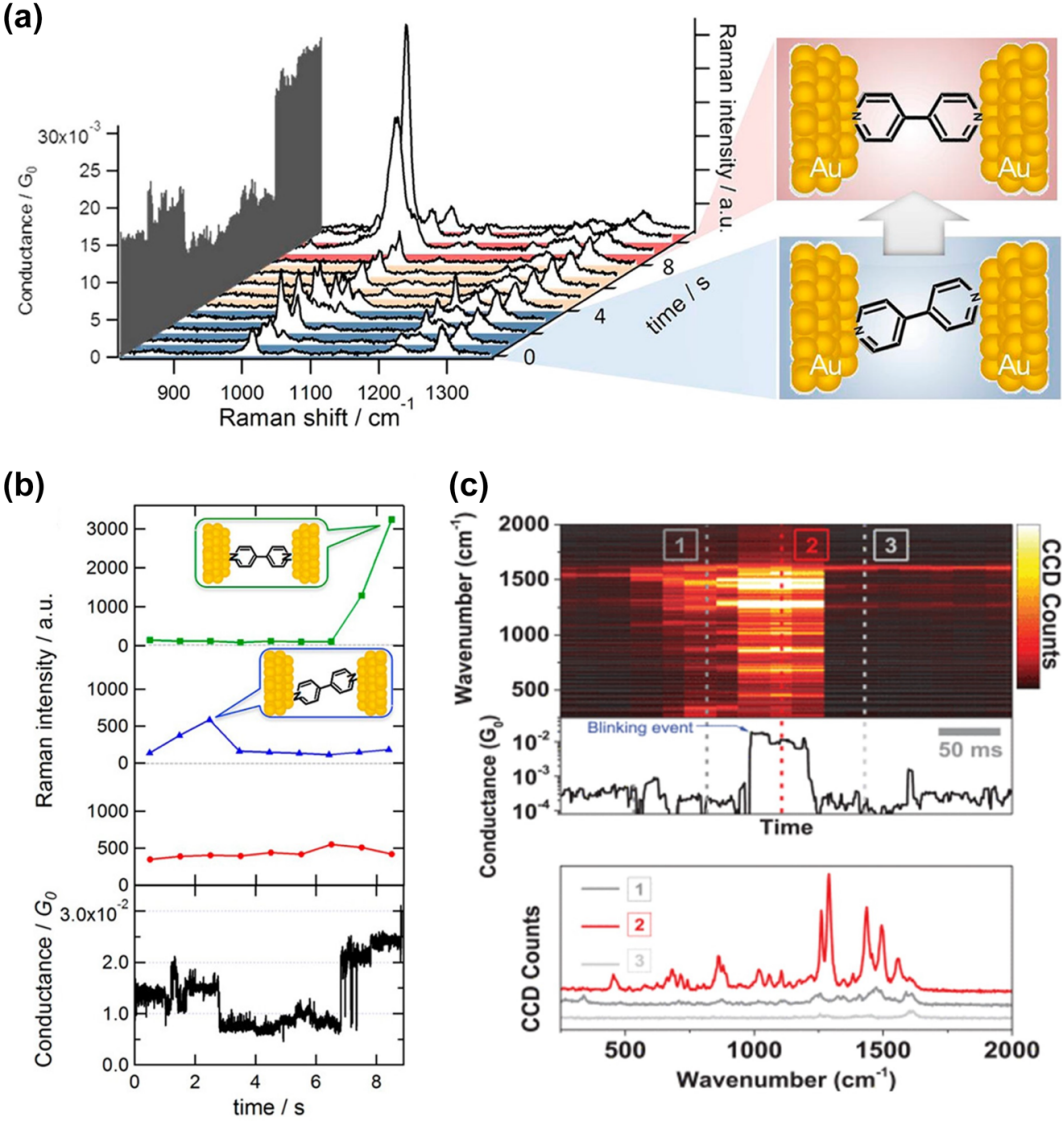
Molecule conductance measurements through Raman sensing. (a) Structure of 4,4′-bipyridine and corresponding Raman spectrum. (b) Raman intensity of a mode (red), b1 mode (blue), and b2 mode (green) together with the conductance of the molecular junction. (c) Correlated Raman spectra and molecular conductance of the BPDT molecule. Raman spectra at three different times denoted as 1, 2 and 3 are depicted in the bottom plot. Figure (a–c) reprinted with permission from Refs. [179], [180].

Konishi et al. performed simultaneous measurements of conductance and surface-enhanced Raman scattering (SERS) signals on 4,4′-bipyridine SMJ in solution at room temperature using the MCBJ technique [179]. The different mode switching in SERS figure is used to figure out the configurations of 4,4′-bipyridine (Figure 18a). The result shows that the configuration change of molecule causes a variation in the energy of different Raman modes, which leads to a switching in the conductance of the MJ (Figure 18b).

Besides, Jeong et al. designed a break junction platform based on the microelectromechanical system (MEMS) [180]. They reveal the charge transport properties of molecules by combining real-time Raman spectroscopy and molecular conductance measurements on this device (Figure 18c).

**Figure 19:**
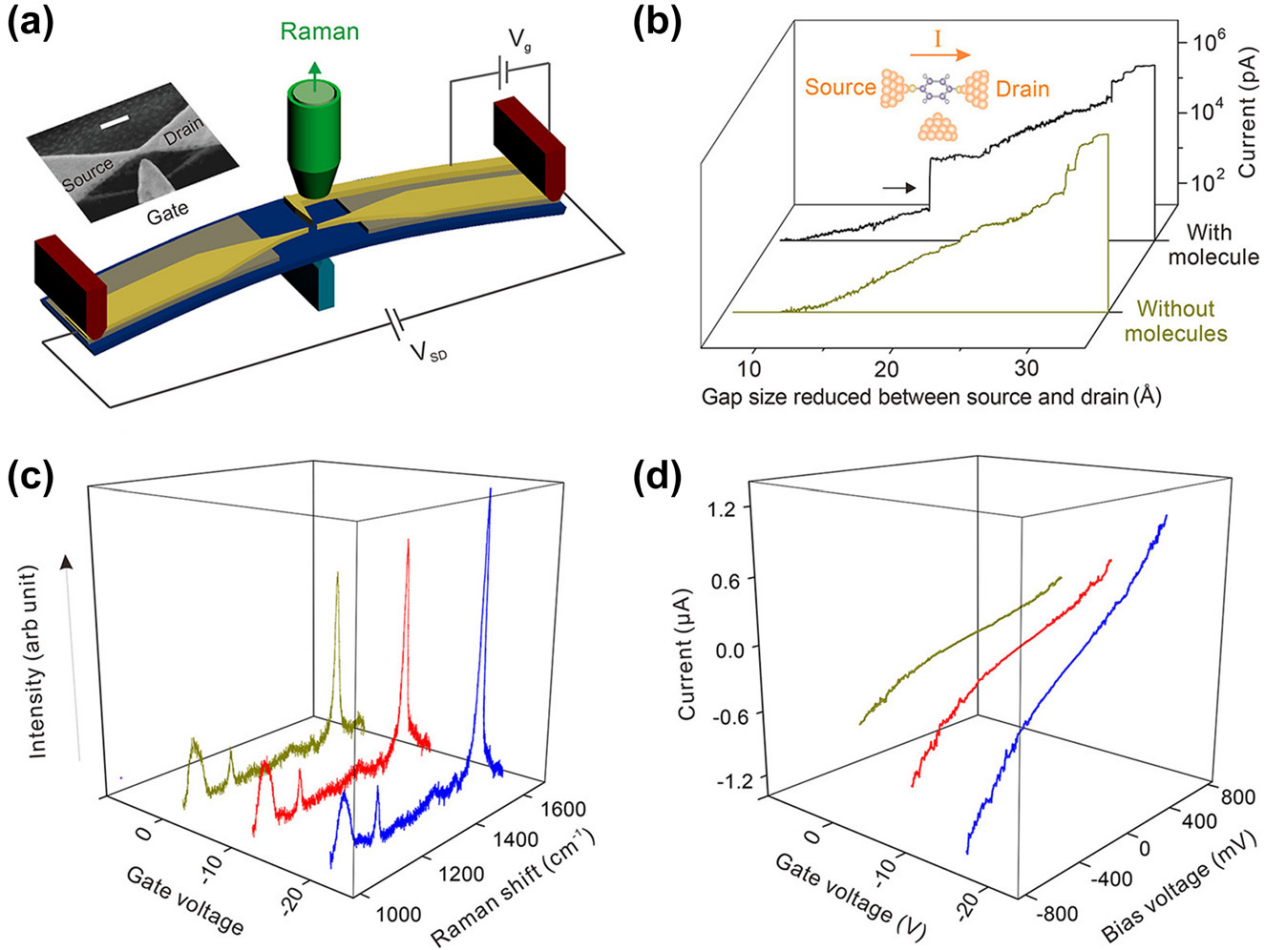
Modified Raman sensing system. (a) Schematic of the modified side-gating Raman scattering system. Inset: SEM image of side-gate electrode. (b) Current traces with and without molecules between electrodes. (c) SERS spectra intensity and *I*–*V* characteristics of 1,4-benzenedithiol molecule junctions upon different gate voltages. Figure (a–d) reprinted with permission from Ref. [181].

To improve the sensitivity of Raman scattering, different aspects which can affect the Raman response have been studied. Modified on MCBJ technique, Guo et al. create a field-effect Raman scattering (FERS) device based on a single molecule in two-terminal MJ, incorporating an additional electrode as a gate (Figure 19a) [181]. When there is current jumping as shown in Figure 19b, it indicated that single molecule junction is formed. Because of the gate effect, when the gate voltage decreases from 0 V to −20 V, the Raman intensity of 1,4-benzenedithiol junction increased a lot and the conductance increased as well (Figure 19c and d). Further DFT calculation and measurement results proved that the intensity of Raman scattering can be amplified by 40 % more than the maximum achievable through electromagnetic enhancement. This enhancement is accomplished through the electrostatic gating of molecular orbitals.

This section presented several innovative techniques that combined Raman spectroscopy with MJ measurement methods. These approaches are adept at directly observing configuration change, chemical reaction, and molecule-electrode coupling in MJ systems. It is expected that these techniques will be widely adopted in future MJ measurements to provide a comprehensive understanding of molecular behavior.

## 7 Conclusion and outlook

Molecular junctions have emerged as an exceptionally productive platform for exploring physical phenomena at the molecular scale, particularly facilitating the direct study of light–matter interactions at the single-molecule level. During the past years, there has been a surge in interest in how light illumination affects charge transport in MJs, along with various emerging effects related to this interaction. This review has methodically examined recent advances in investigating the optoelectronic properties of illuminated MJs, encompassing a diverse range of molecular species in both single-molecule and SAM configurations. Additionally, progress in molecular-scale electronics featuring key photoswitchable molecules like azobenzene, diarylethene and dihydropyrene has been summarized. These Molecular-scale devices have shown optoelectronic behaviors similar to or even better than traditional semiconductor materials with high on/off ratio or prominent conductance property, as proven in experiments. These advancements in understanding and technology are foundational for the development of future molecular optoelectronic components.

Despite this progress, some challenges remain need to be addressed. First, creating high-performance molecular devices with high on/off ratios requires strategic molecular design. For example, different C_60_ structure in the decanethiol compound system will perform different conductance property. Additionally, observed differences between single-molecule and self-assembled monolayer junctions comprising the same molecule warrant investigation to understand these discrepancies. In the case of SAM-based junctions, electrode requires high optical transmittance material and steric hindrance effect prevent the isomerization reaction of photoswitchable molecules. This insight is essential for the design of larger-area devices using molecular assembly techniques. Furthermore, there is a pressing need to explore novel molecular junction device structures and architectures, including the integration of supramolecules, polymers, and plasmonic nanostructures into MJs. Finally, accurately simulating the transmission characteristics of MJs and their response to light illumination remains a significant challenge. For instance, the conductance of the trans-cis isomerization in one molecule shows opposite results. Some experiments indicate that the cis isomers present higher conductance [56], [114], [182] while some article found that the trans isomers show higher conductance [115], [142], [164], [183]. This conflict is caused by different factors such as conformation of the molecule, anchoring group, electrodes coupling and so on, which is intriguing and requires further investigation. Besides, the photoisomerization mechanism in the DHP/CPD system is still under active discussion. Addressing these challenges is key to advancing the field of molecular electronics and developing more sophisticated and functional molecular-scale devices.
